# Characterization of electrochemical deposition of copper and copper(I) oxide on the carbon nanotubes coated stainless steel substrates

**DOI:** 10.1038/s41598-023-33963-w

**Published:** 2023-04-26

**Authors:** Jakub Marchewka, Ewa Kołodziejczyk, Patryk Bezkosty, Maciej Sitarz

**Affiliations:** Faculty of Materials Science and Ceramics, AGH University of Science and Technology, Al. Mickiewicza 30, 30-059 Kraków, Poland

**Keywords:** Chemistry, Materials science, Nanoscience and technology

## Abstract

The nanocomposite coatings composed of carbon nanotubes and various forms of copper were prepared in the two-step process. Firstly, carbon nanotubes were coated on stainless steel substrate using electrophoretic deposition at constant current. Then, the process of electrochemical deposition using copper(II) sulphate solutions was performed under high overpotential conditions. The modification of the copper(II) cations concentration in the solution and the deposition time provided the formation of various forms of crystals. The samples and their cross-sections were observed and examined using scanning electron microscope equipped with electron dispersive spectroscopy system. The analysis of chemical composition revealed that in addition to the pure copper crystals, the crystals characterized by the presence of copper and oxygen were formed. Therefore, Raman spectroscopy was applied to determine the unknown stoichiometry of this copper oxide. The point and in-depth analysis identified copper(I) oxide in the form of different size crystals depending on the concentration of the copper(II) sulphate solution. To confirm these findings, grazing incidence X-ray diffraction measurements were also performed. the combination of the applied methods has provided the detailed description of the preparation of the nanocomposite coatings with the proposed mechanism of copper(I) oxide formation.

## Introduction

Electrochemical methods are still considered one of the most favorable techniques of production of composite coatings, despite the continuous development of other methods [such as physical vapor deposition (PVD) or chemical vapor deposition (CVD)]. They stand out from the rest because of their high versatility, repeatability and simplicity in combination with precise control over the properties of obtained products^1,2^. The other advantages are low cost, reduced amount of waste materials and easy scalability of the equipment used in electrochemistry processes. Moreover, by changing the process parameters, it is possible to tailor the crucial properties of coatings such as their thickness, roughness and morphology^3–5^. These advantages, together with variety of received materials and their applications place electrochemistry in the spotlight for researchers from many fields of science. Composite coatings, wherein the particles in the conductive matrix could be metallic, polymeric or ceramic have been used successfully in electronics, surface engineering, aerospace or corrosion protection^6–11^. Electrochemical deposition (ECD) processes have been known since the beginning of the nineteenth century, but research is still ongoing to explain their mechanisms. Although, electrochemical reactions occurring during the electrodeposition process are relatively easy to balance by several redox equations, the individual steps of the process which run according to specific mechanisms are still the subject of research and process modeling^12,13^. To put it simply, the ECD is based on the modification of the conductive substrate surface with a thin and tight adherent coating of desired material deposited from solution. It takes place at the interface of the two phases: liquid (electrolyte) and solid (electrodes) in the closed electrical circuit. The system could be far from the state of chemical equilibrium, because the applied potentials may differ from the equilibrium values determined by the Nernst equation or the Pourbaix diagrams^14^. Therefore, by controlling the applied potential and pH, it is possible to obtain various forms (chemical, structural or crystallographic) of material from the same solution^15–17^.

Copper is one of the metals most widely used in the industry and currently most applied material in ECD. This is mainly due to its excellent thermal and electrical conductivity and anti-corrosion properties. In acidic solutions, copper(II) cations (Cu^2+^) are directly reduced to metallic copper (Cu) according to the following reaction:

$${{Cu}^{2+}}_{(aq)}+{2e}^{-}\to {Cu}_{(s)}$$

Metallic coatings of copper with appropriate thickness are characterized by structure free of porosity and good adhesion^18,19^. Thin films of electrodeposited copper have been studied extensively in connection to their application in microelectronics, aerospace, gas-sensors, superhydrophobic materials, surface protection or even additive manufacturing^20–25^. However, metallic copper is not always a product of this electrochemical reaction. Due to the water reduction, we can observe local alkalization between the solution and electrode. Under these conditions other forms of copper may be formed. As it is described in the Pourbaix diagram for copper in aqueous system, with use of an appropriate overpotential copper oxides—CuO and Cu_2_O—could be formed^26,27^.

Furthermore, by the appropriate control of the deposition time, we are able to obtain crystals of copper and its oxides of various shapes and geometry. It is strictly related to the mass transport (ion diffusion to the electrode) and kinetics of the electrochemical reaction, which are the stages limiting the deposition rate of whole process^28,29^. It was demonstrated that copper oxides obtained in a simple electrodeposition route by changing only a few parameters (e.g., potential, pH or deposition time) may have nanometric dimensions^30^.

In the literature, efforts have been made to theoretically understand the mechanisms of copper electrodeposition^31–33^. In turn, in the latest research, articles focusing on potential applications of composite materials containing copper and its oxides obtained electrochemically lead the way^34–37^. Therefore, in this work we would like to place special emphasis on the characterization of ECD on the carbon nanotubes coated stainless steel using copper(II) sulphate solutions. Combination of copper and carbon nanotubes in one composite coating obtained by ECD has been the subject of research in recent years in relation to high mechanical, tribological and electrical properties of those materials^38,39^ and their potential applications, including glucose sensor or anode in Li-on batteries^40,41^. In this work, we aim to show the influence of deposition parameters and the presence of carbon nanotubes layer on the mechanism of the formation of various structures of copper and copper(I) oxide.

## Materials and methods

### Preparation of the stainless steel plates

All the samples were prepared using austenitic chromium-nickel steel AISI 304 (1.4301 according to the EN10088-1 standard) in the form of 10 × 20 mm plates as a substrate. Before the deposition of carbon nanotubes (CNT) layer the mechanical and chemical cleaning was performed. It included the following steps: abrasive sandpaper grinding (600 grit), sonication in acetone (≥ 99.5%, Avantor Performance Materials, Poland) and then in ethanol (≥ 96%, Avantor Performance Materials, Poland) for 5 min each using the ultrasonic bath (Sonic-0.5, Polsonic, Poland). Cleaned plates were stored in ethanol and dried just before the preparation of CNT layer.

### Chemical oxidation of CNT

Multi-walled CNT (CNT Co. Ltd., South Korea) with a diameter in the range of 5–20 nm and a length of about 10 µm were chemically oxidized in the 3:1 (v/v) mixture of concentrated sulfuric acid (≥ 95%, Avantor Performance Materials, Poland) and concentrated nitric acid (35–38%, Avantor Performance Materials, Poland). For this, they were treated in the solution under a reflux condenser for 4 h at 70 °C and then washed with distilled water and dried. The process was performed to introduce the negatively charged carboxyl (COO^−^) functional groups into the surface of CNT, as well as carbonyl (C=O) groups and ether (C–O–C) moieties^42^. Therefore, the resulting negative zeta potential made possible to conduct their electrophoretic deposition (EPD) on the substrate as an anode. Furthermore, the remnants of metallic catalysts from the CNT synthesis could been removed during the process.

### Preparation of CNT layers

CNT after chemical oxidation were added to the 2:1:1 (v/v/v) mixture of acetone (≥ 99.5%, Avantor Performance Materials, Poland), ethanol (99.8%, Avantor Performance Materials, Poland) and distilled water. The 1 mg/mL solution was sonicated for 5 min using Sonic-0.5 (Polsonic, Poland) ultrasonic bath to improve the CNT dispersion. The process of EPD of CNT on stainless steel plates was performed using the custom-made equipment with PLH250 (Aim-TTi, United Kingdom) DC power supply. Three electrodes arrangement was applied including two counter electrodes with a distance of 15 mm as cathodes and the substrate mounted in a holder symmetrically between them as an anode. The CNT solution was poured into a polypropylene vessel prepared by Fused Deposition Modeling (FDM) 3D printing. For this, 1.75 mm polypropylene filament (Verbatim, Japan) and UBOT 3D S+ (UBOT 3D, Poland) 3D printer were used. The vessel dimensions were designed to fit to the geometry of the applied electrodes arrangement and therefore to reduce the consumption of the solutions. All CNT layers were prepared in the process of EPD using the voltage of 30 V for 60 s. Then, the samples were dried in a room temperature.

### Preparation of the samples

Copper(II) sulfate (≥ 99.99%, Sigma-Aldrich, USA) solutions in distilled water were prepared with the concentration of 1 mM and 100 mM. The process of electrochemical deposition (ECD) on the CNT-coated stainless steel plates was performed using the same equipment as for EPD. As a difference in the arrangement the two counter electrodes performed as anodes whereas the CNT-coated stainless steel plate as a cathode. The samples were prepared using 1 mM or 100 mM CuSO_4_ solution in the process of ECD with the application of the voltage of 7.0 V for 60 s, 120 s, 180 s, 240 s, 300 s, 450 s or 600 s. Then, they were washed with distilled water and dried in a room temperature. Two series of the samples including A1–A7 and B1–B7, respectively, were obtained (Table 1).

**Table 1:** The summary of the samples prepared in the process of ECD.

Sample	CuSO_4_ concentration (mM)	Deposition time (s)	Sample	CuSO_4_ concentration (mM)	Deposition time (s)
A1	1	60	B1	100	60
A2	1	120	B2	100	120
A3	1	180	B3	100	180
A4	1	240	B4	100	240
A5	1	300	B5	100	300
A6	1	450	B6	100	450
A7	1	600	B7	100	600

### SEM–EDS characterization of the samples

The microstructure and chemical composition of the samples were investigated using Phenom XL (Thermo Fisher Scientific, USA) scanning electron microscope (SEM) equipped with electron dispersive X-ray spectroscopy (EDS) system. The examination included the surfaces and the cross sections. Back-scattered electrons detector and the acceleration voltage of 10 kV (for SEM images) or 15 kV (for EDS analysis) were applied. The characterization of the cross sections required their appropriate preparation. The applied procedure included the following steps: the deposition of a thin gold coating (about 20 nm) to improve the adhesion of an additional nickel layer to the investigated material using EM ACE200 (Leica, Japan) vacuum coater, the application of a protective nickel coating (with the thickness of about 2–3 μm) by electroplating technique, the immersion of the samples in a temperature resistant resin (Struers, Denmark) and finally their polishing according to the standard Struers procedure.

### Raman spectroscopy analysis of the samples

The structure of the samples was analyzed by Raman spectroscopy using alpha300 M+ (WITec, Germany) spectrometer equipped with a confocal microscope. All measurements were performed with the application of 488 nm diode laser and 600 grating. The characteristic features observed on the surface of the samples were examined by single point analysis. For this, four accumulations with the integration time of 20 s were collected. The in-depth analysis (Z line scans) was performed to evaluate the inner composition of CNT layers. The 4.0 μm depth profiles with a step of 0.5 μm were characterized by collecting four accumulations with the integration time of 20 s in each step. For the comparison the analysis for pure copper oxides including Cu_2_O (≥ 99.,99%, Sigma-Aldrich, USA) and CuO (≥ 99.99%, Sigma-Aldrich, USA) were performed. For this, three or six accumulations, respectively, with the integration time of 20 s were collected. The data was processed using Bruker OPUS 7.2 software.

### GIXRD measurements of the samples

Phase composition of the samples was characterized by grazing incidence X-ray diffraction (GIXRD) measurements using Empyrean (Malvern Panalytical, United Kingdom) diffractometer with CuKα1 line applied as a radiation source and Ge(111) as a monochromator. The scans were collected applying θ–2θ geometry in 2θ range from 10 to 90° and 0.001° step size with an incident angle of 1.0°. The data was processed using Highscore Plus 3.0 software with PDF-4+ database.

## Results and discussion

### The scope of the tests

The samples were obtained in the form of CNT layers on the stainless steel plates modified by the process of ECD using CuSO_4_ solutions. Their examination was focused on the microstructure and composition of the electrodeposited material in relation to the parameters of ECD process. Significantly different concentrations of CuSO_4_ solutions, i.e. 1 mM and 100 mM, as well as high overpotential conditions were intentionally applied. A general overview of the samples with particular attention to the form of the electrodeposited material on the surface was carried out using SEM. This method was also applied for the cross sections of the selected samples to examine the microstructure of CNT layers. During this analysis, the elemental composition was evaluated by EDS with a special emphasis on copper and oxide. Two samples (A7 and B7) prepared by the longest running process of ECD (600 s) were additionally investigated in detail as the most complex systems in terms of the microstructure. Raman spectroscopy was applied to examine the distinctive forms of the electrodeposited material using the point and in-depth analysis. To confirm the results of the tests the GIXRD measurements were also performed.

### Material electrodeposition in terms of the process parameters

The initially applied EPD method enables the preparation of the tight CNT coatings on the stainless steel substrates. In the process of ECD two regions for the material electrodeposition could be distinguished. The first includes the surface of CNT layer whereas the second one is its volume. Therefore, they were analyzed separately in this research. At the beginning, the selected and applied ECD process parameters should be considered. All samples were prepared in high overpotential conditions. It is known^43–45^ that this parameter is critical for the reduction rate of metal cations in similar systems. In order to understand this phenomenon two parts of the solution volume could be distinguished, i.e. a thin interface directly in the vicinity of a substrate and a bulk solution. When a low overpotential is applied Cu^2+^ cations are relatively slow depleted from the near substrate layer. Consequently, their concentration is easily replenished and maintained by their diffusion from the bulk solution. Crystal formation is limited by the process of Cu^2+^ cations reduction and driven by the minimization of surface energy. Contrary, when high overpotential is applied the deficiency of Cu^2+^ cations in the proximity of the substrate is relatively rapidly generated. Therefore, their diffusion rate from the bulk solution is not enough to maintain their concentration in this volume. Crystal growth is limited by the process of mass transport. It could be also mentioned that high overpotential conditions in water solutions promote the evolution of hydrogen on an electrode^46–48^. This effect is important when the aim is to prepare a tight layer of electrodeposited material on a substrate. As a consequence of the production of gas bubbles and their diffusion from the substrate surface to the bulk solution the cavities are formed. It was not observed for the prepared samples, because the duration of ECD process was too short to obtain a high surface coverage by the electrodeposited material. As it is described in the Pourbaix diagram for Cu^26,27^ the potential which provides the reduction of Cu^2+^ cations to metal Cu is about hundreds of mV depending on the pH of the solution. Therefore, in this research high overpotential conditions were applied. This results in the domination of the mass transport limited mechanism of the crystals formation.

### Characterization of the samples surface by SEM

**Figure 1:**
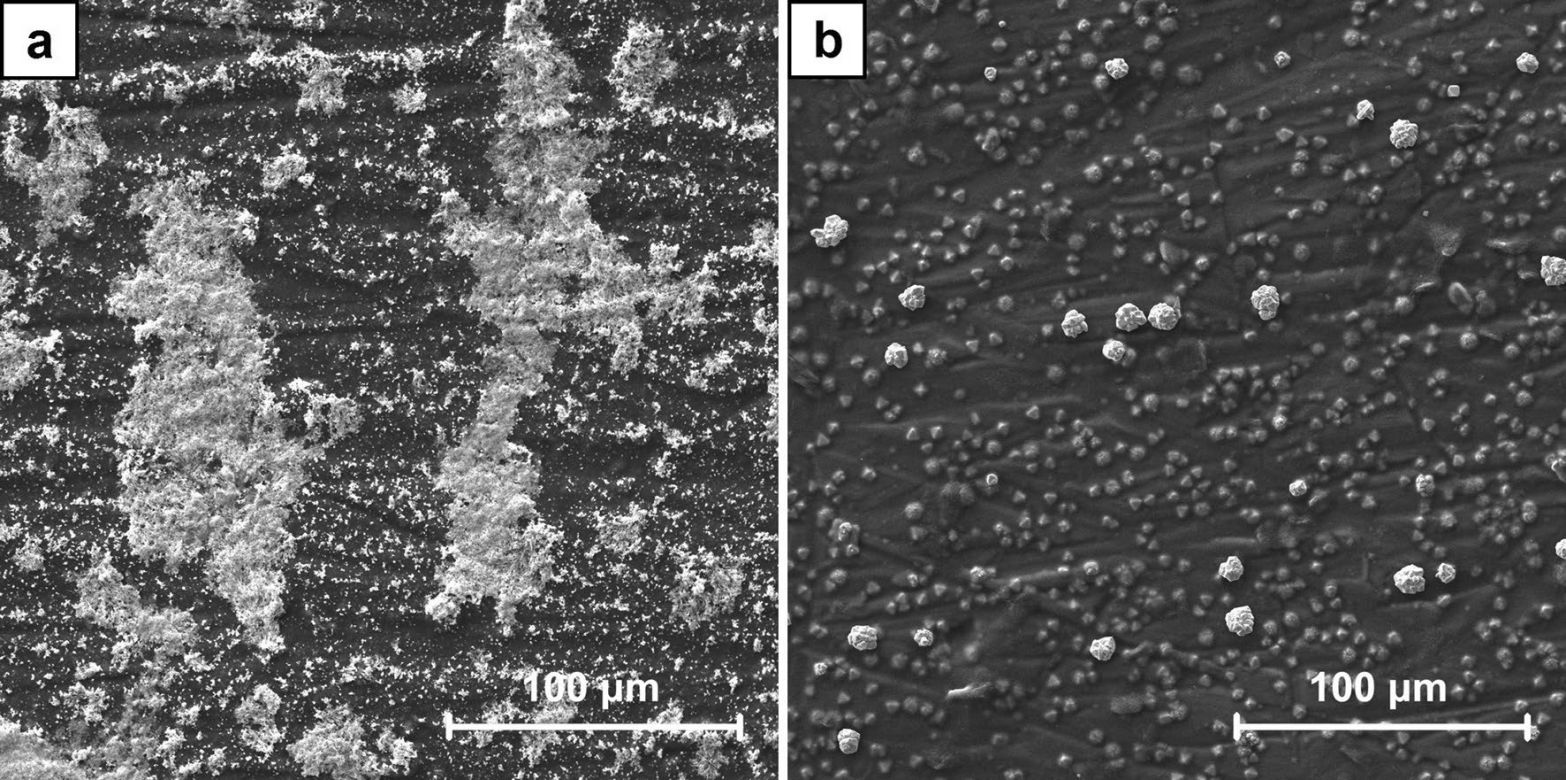
The comparison of SEM images of A7 sample (ECD deposition time of 600 s, CuSO_4_ concentration of 1 mM) (**a**) and B7 sample (ECD deposition time of 600 s, CuSO_4_ concentration of 100 mM) (**b**) at the magnification of ×1000.

**Figure 2:**
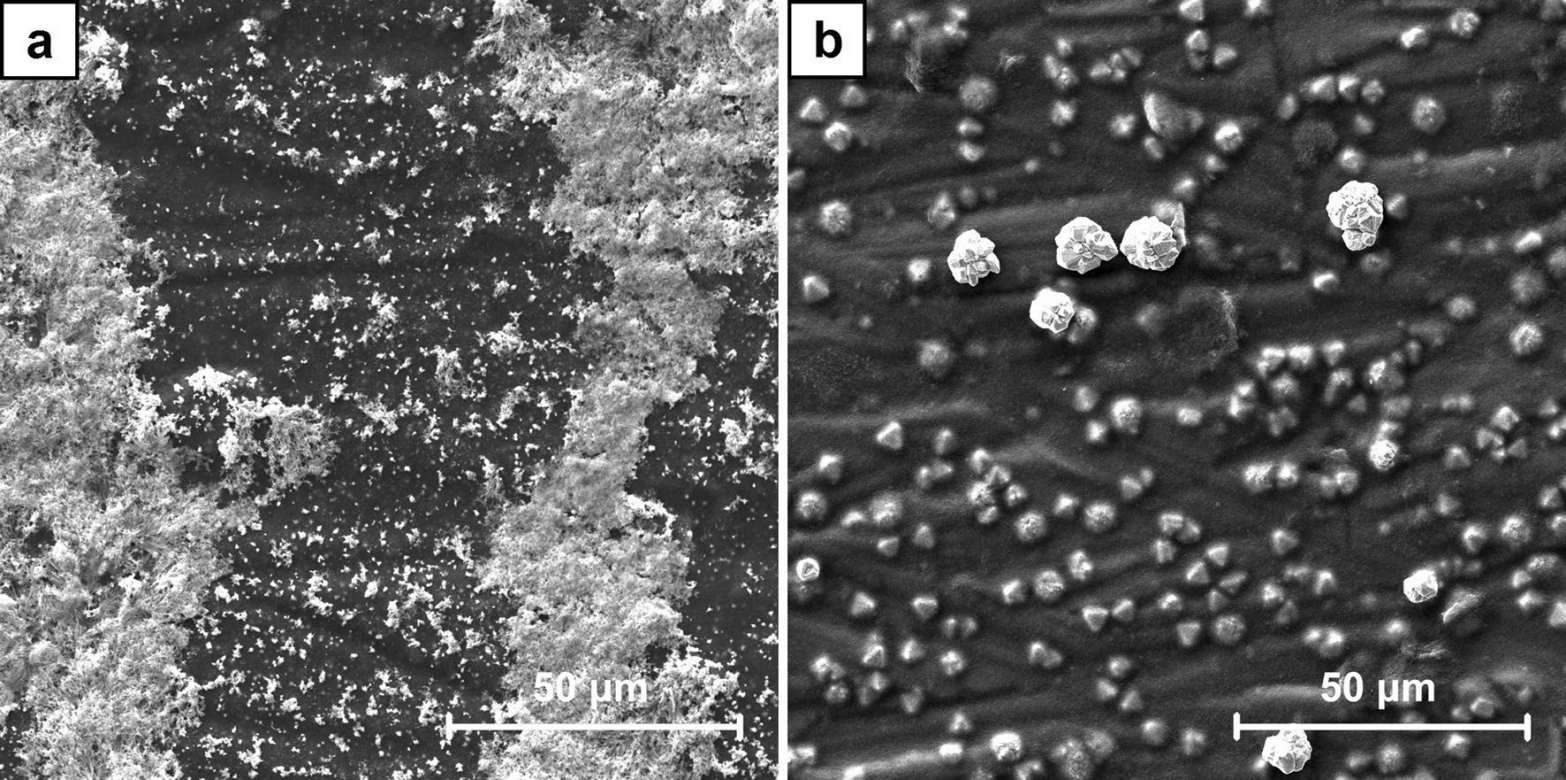
The comparison of SEM images of A7 sample (ECD deposition time of 600 s, CuSO_4_ concentration of 1 mM) (**a**) and B7 sample (ECD deposition time of 600 s, CuSO_4_ concentration of 100 mM) (**b**) at the magnification of ×2000.

**Figure 3:**
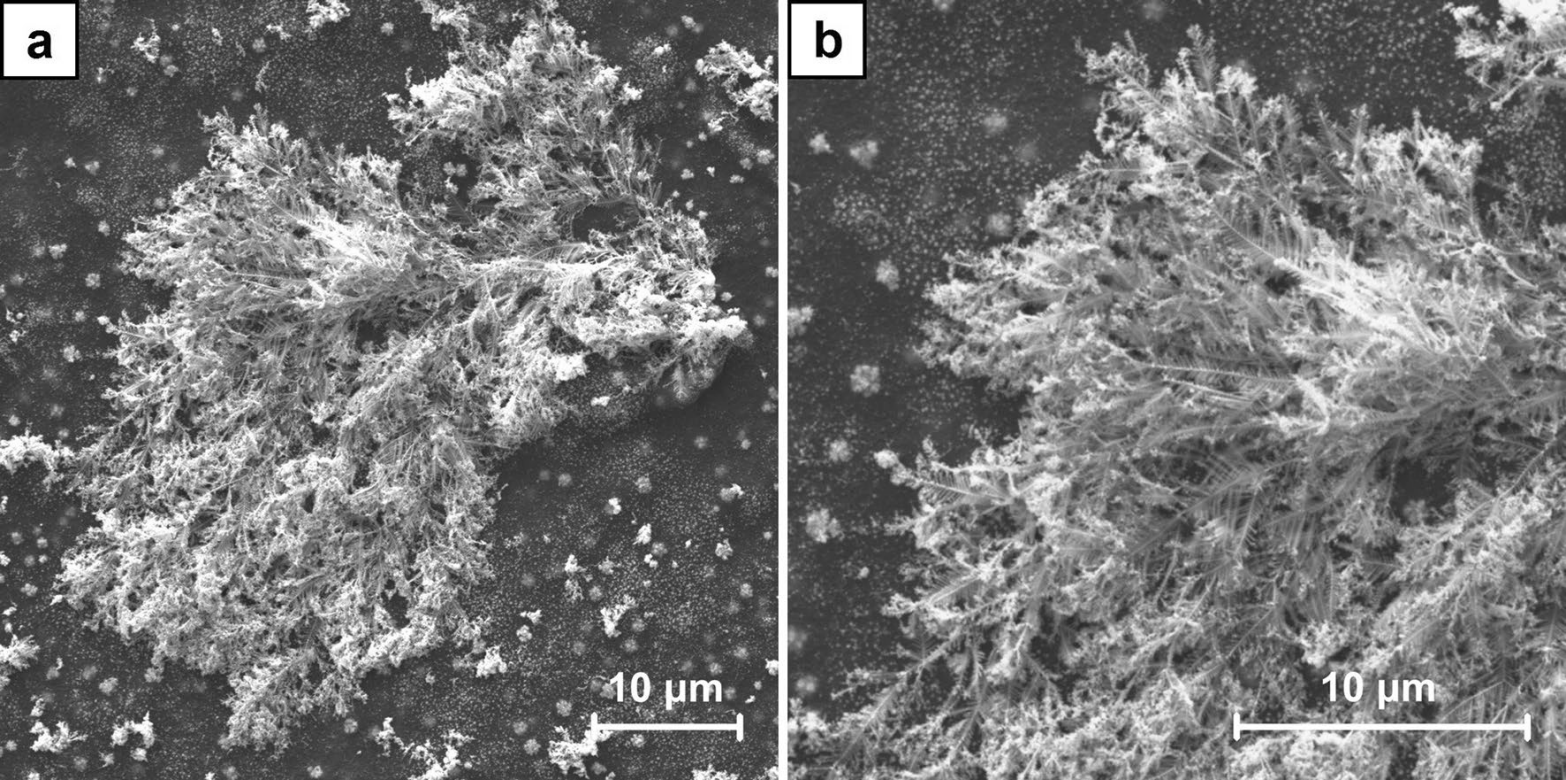
SEM images of the dendritic crystals formed on CNT layer in A6 sample (ECD deposition time of 450 s, CuSO_4_ concentration of 1 mM) at the magnification of ×5000 (**a**) and ×10,000 (**b**).

**Figure 4:**
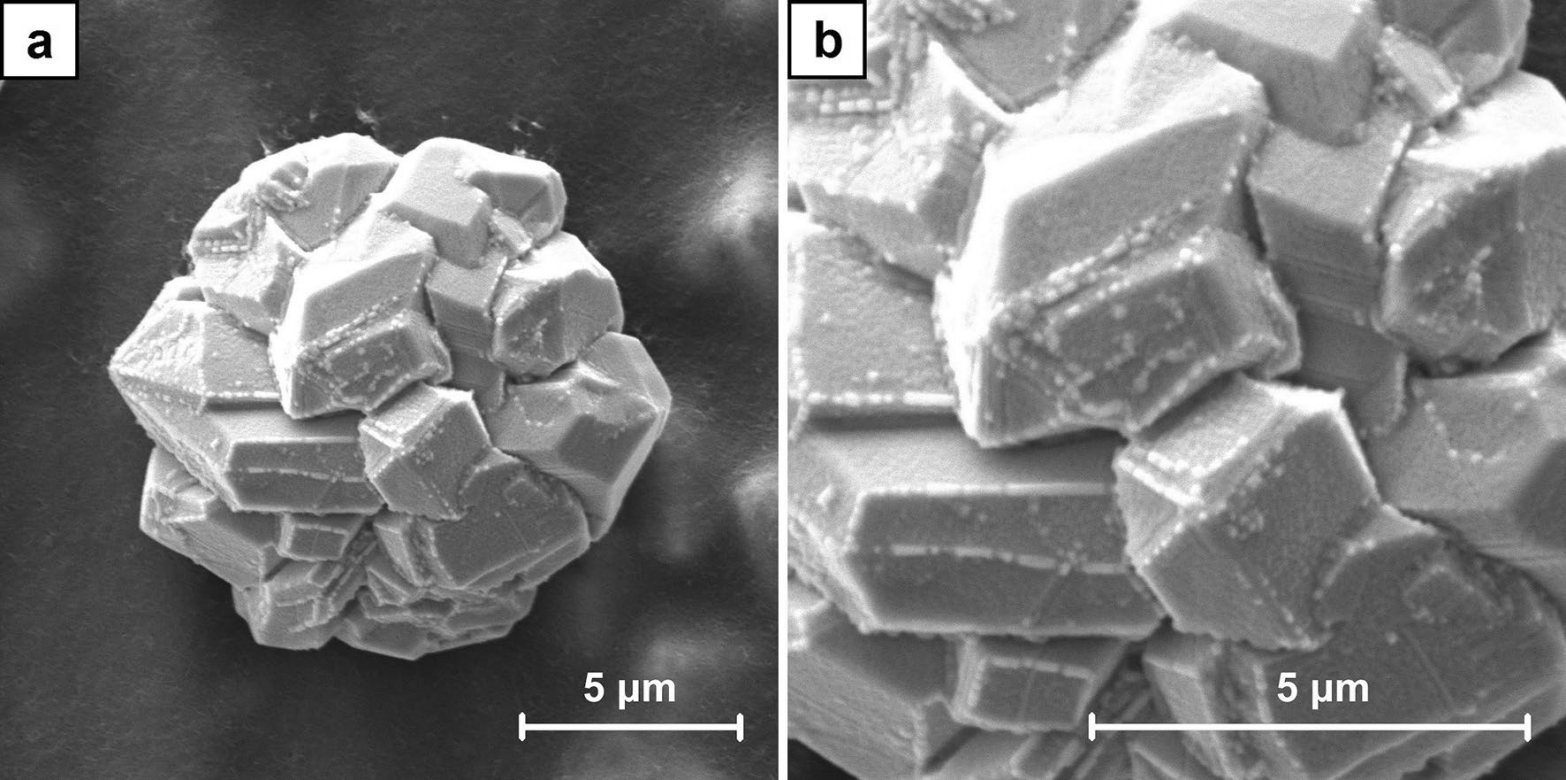
SEM images of the crystals agglomerate formed on CNT layer in B7 sample (ECD deposition time of 600 s, CuSO_4_ concentration of 100 mM) at the magnification of ×15,000 (**a**) and ×30,000 (**b**).

Two series of the samples were prepared in the process of ECD using the different concentration of CuSO_4_ solutions, i.e. 1 mM for A1–A7 and 100 mM for B1–B7. Hence, with the application of the same high overpotential the concentration of Cu^2+^ cations play a major role in the mechanism of the crystals growth. As it is shown in the SEM images of A7 and B7 samples (Figs. 1, 2) a significantly different crystal structures formed on CNT layers. Using 1 mM solutions in the process of ECD the dendritic crystals were obtained (Fig. 3). These structures are typically formed in a local non-equilibrium state^49–52^. Hence, in this case the typical mechanism of crystal growth limited by the mass transport may be assumed, as it was explained before. Contrary, using 100 mM CuSO_4_ solutions the spherical crystals agglomerates were produced (Fig. 4). Well-defined single crystals are formed when the mechanism of their growth is driven entirely by the kinetics of the process of cations reduction. The observation of the agglomerates could indicate the existence of mixed mechanism limited partially by both the mass transport as well as the Cu^2+^ cations reduction. The effect resulting from the application of high overpotential conditions was partially reduced by a significantly faster diffusion rate of Cu^2+^ cations from the bulk solution to the interface. This was provided by the significantly higher concentration of Cu^2+^ cations used for the preparation of B series samples in comparison to A series samples.

**Figure 5:**
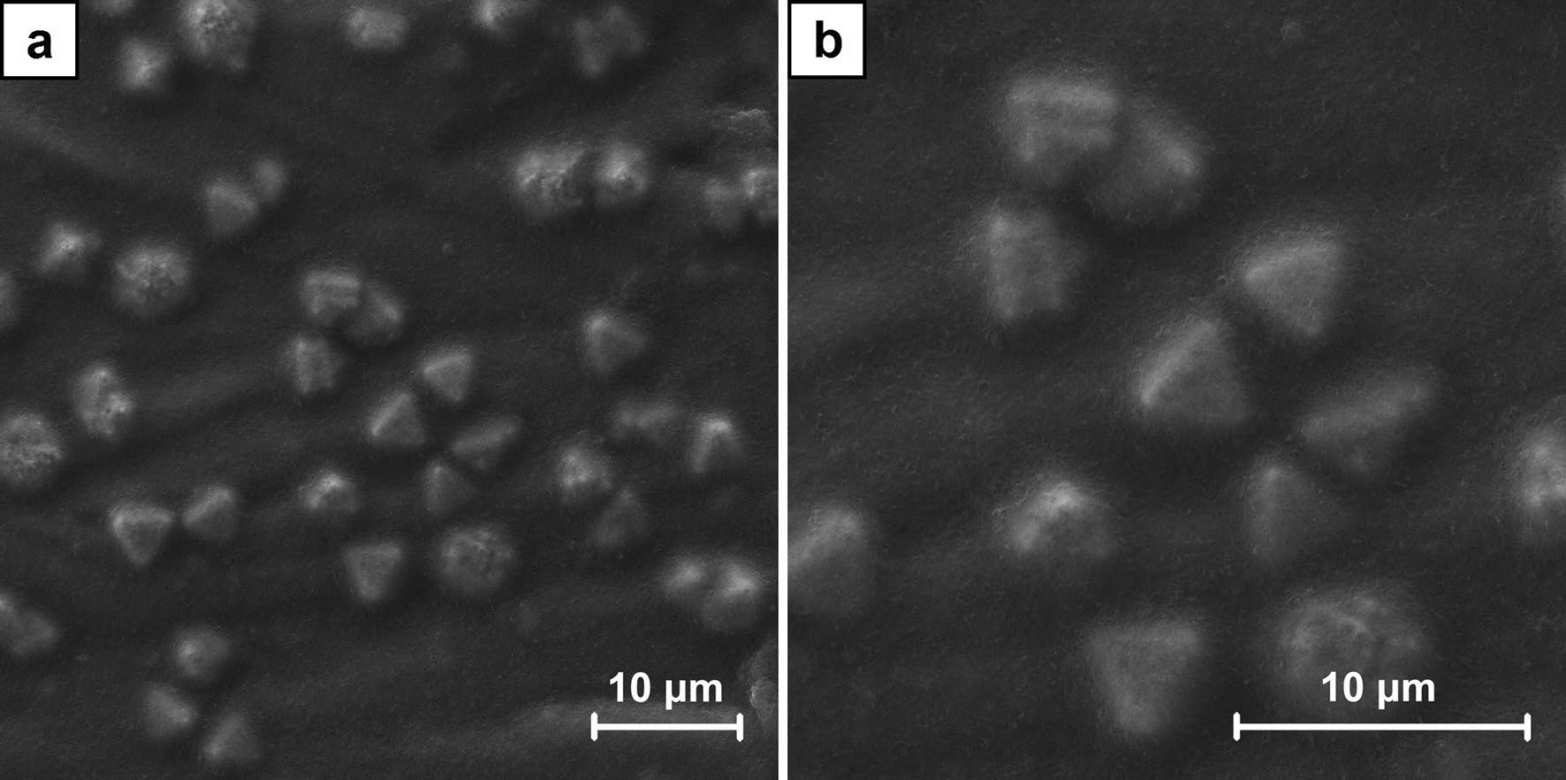
SEM images of the crystals which formed within CNT layer in B6 sample (ECD deposition time of 450 s, CuSO_4_ concentration of 100 mM) at the magnification of ×5000 (**a**) and ×10,000 (**b**).

**Figure 6:**
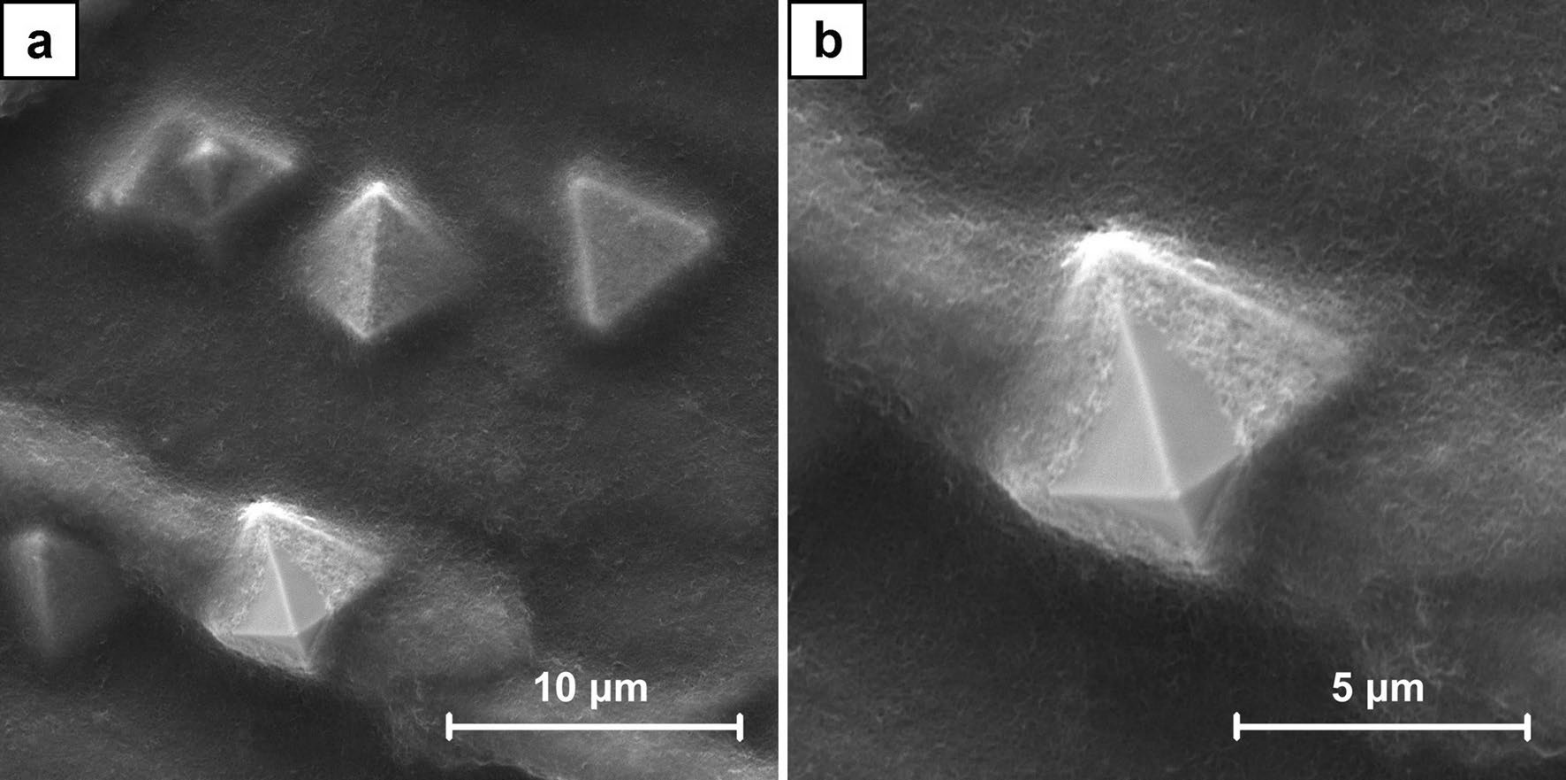
SEM images of the crystals which grew to the surface of CNT layer in B7 sample (ECD deposition time of 600 s, CuSO_4_ concentration of 100 mM) at the magnification of ×10,000 (**a**) and ×20,000 (**b**).

To examine the crystal growth two series of the samples were prepared. The SEM images (Figs. S1–S7 and Figs. S8–S14, respectively, in supplementary data) reveal their gradual formation. For A series samples the first well-defined dendrites were observed after 120 s of the electrodeposition (Fig. S2). With the increasing duration of the process, they progressive growth was revealed and the surface coverage was higher. The examination of B series samples shows a significantly different picture. The formation of the spherical agglomerates is already noticed after 60 s of the electrodeposition (Fig. S8). They are growing bigger with time. The other effect is the formation of crystals within CNT layer. At the beginning they are small and could be noticed as its local inhomogeneity (Fig. S8), but with the increasing duration of the process they are also growing bigger. Finally, some of these crystals get a spherical shape, but the other obtain a well-defined octahedral shape (Figs. 5, 6). It was also found that after 600 s of the electrodeposition the octahedral crystals grow to the surface of CNT layer.

### Analysis of the crystals structure by Raman spectroscopy

**Figure 7:**
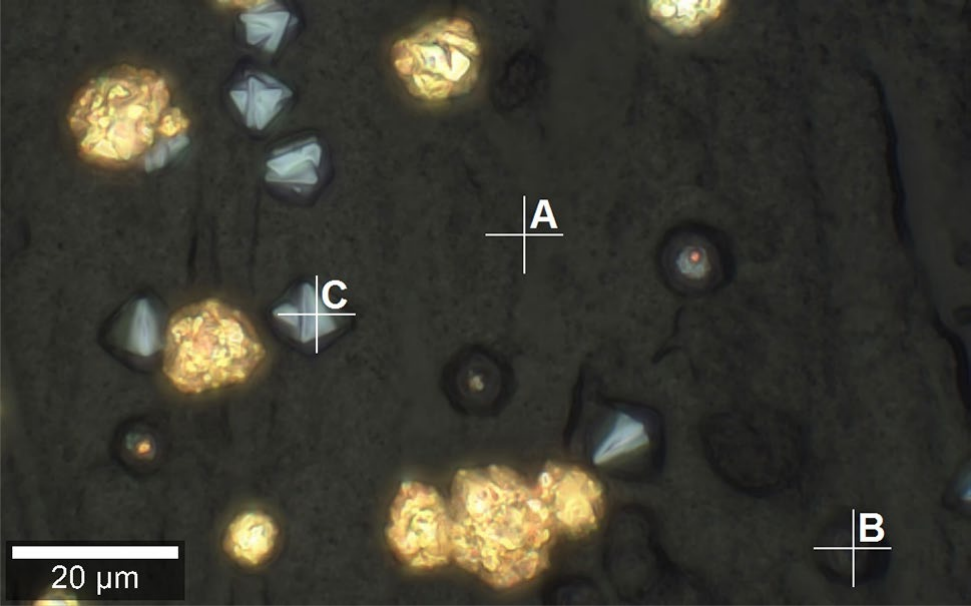
Confocal microscope image of B7 sample (ECD deposition time of 600 s, CuSO_4_ concentration of 100 mM) for the single point Raman analysis.

**Figure 8:**
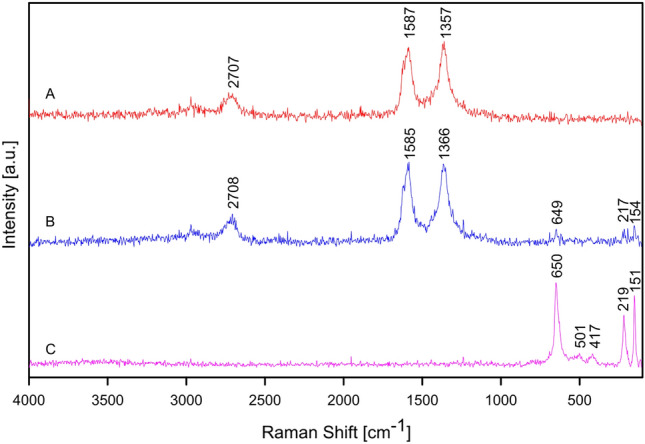
Raman spectra of the B7 sample (ECD deposition time of 600 s, CuSO_4_ concentration of 100 mM) in points A–C.

Raman spectroscopy was used to examine the formation of crystals on CNT layer (Fig. 7). For this, the single point measurements were performed for B7 sample in the characteristic points chosen using confocal microscopy (Fig. 8). The first one is positioned on pure CNT layer (point A) whereas the others correspond to the different crystal structures, including the spherical agglomerate which partially grew to the surface (point B) and the octahedral crystal on the surface (point C). In this microphotography the spherical Cu agglomerates are also noticed. Copper, like every metal, is not active in Raman spectroscopy, so the appropriate spectra are not obtained. The first spectrum (for point A) is typical for CNT layer on the stainless steel substrate as we reported previously^53,54^. Two the most intense bands are located at 1357 cm^−1^ (so called D band) and at 1587 cm^−1^ (so called G band). The first one is related to the presence of disorder in sp^2^ carbon structure (A_1g_ mode) whereas the second one is associated with the C–C vibrations, both along the CNT axis and circumferential (E_2g_ mode). The other weak band observed at about 2710 cm^−1^ is a second-order harmonic of the D mode (so called 2D band) which could be attributed to the three dimensional long range order of CNT^55,56^. The third spectrum (for point C) was obtained for the octahedral crystal on the surface. A set of bands typical for Cu_2_O at the range from 150 to 650 cm^−1^ are found. This could be confirmed by the comparison (Table 2) with the spectra obtained for pure copper oxides (Fig. S15, in supplementary data). The most intense band at 647 cm^−1^ is associated with the T_1u_(LO) mode. The other ones at 151 cm^−1^ and 219 cm^−1^ are attributed to the T_1u_(TO) and 2E_u_ modes, respectively. Finally, the weak bands at 417 cm^−1^ and 501 cm^−1^ could be related to the four-phonon 3E_u_ + T_1u_(TO) mode and to the T_2g_ mode, accordingly. Therefore, this assignment proves that the observed octahedral crystals are composed of Cu_2_O^57,58^. In case of the measurement for point B, the spectrum is a result of the probing depth characteristic of Raman spectroscopy. The bands at 1366 cm^−1^, 1585 cm^−1^ and 2708 cm^−1^ are associated with CNT which form the layer. They are analogous to those observed in the spectrum obtained at point A. Additional weak bands between 150 and 650 cm^−1^ indicate the presence of Cu_2_O in the crystal structures that are formed within the CNT layer. Their positions are similar to the positions of the most intense and characteristic bands as it was noted in the spectrum obtained at point C. Thus, it can be confirmed that the probing depth made it possible to determine the presence of both CNT and Cu_2_O. No bands characteristic of CuO were observed in the spectrum at any of the points studied^59^.

**Table 2:** The bands observed for Cu_2_O in B7 sample compared with the bands for pure copper oxides.

B7 sample point B bands (cm^−1^)	B7 sample point C bands (cm^−1^)	Cu_2_O reference bands (cm^−1^)	CuO reference bands (cm^−1^)
154	151	147	–
217	219	219	–
–	–	–	292
–	–	–	342
–	417	413	–
–	499	491	–
		500	–
–	–	–	624
650	647	630	–
		643	–

**Figure 9:**
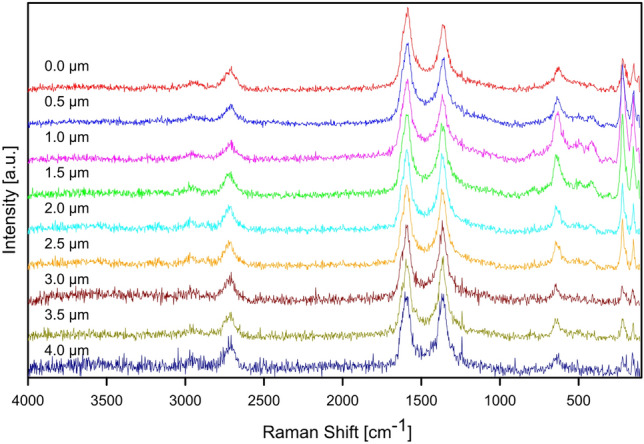
Raman in-depth analysis of the A7 sample (ECD deposition time of 600 s, CuSO_4_ concentration of 1 mM) from the surface to the depth of 4.0 µm.

**Figure 10:**
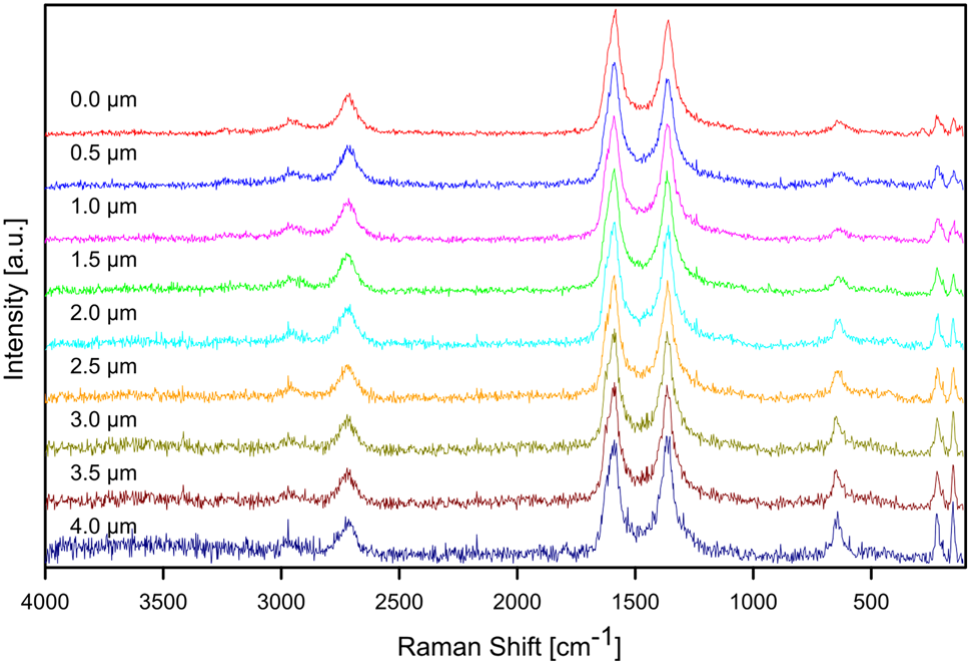
Raman in-depth analysis of the B7 sample (ECD deposition time of 600 s, CuSO_4_ concentration of 100 mM) from the surface to the depth of 4.0 µm.

Raman spectroscopy was also used to investigate the inner composition of CNT layers after the process of ECD. The in-depth analysis was performed for A7 and B7 samples from the surface to the depth of 4.0 µm. In the both sets of spectra (Figs. 9, 10) the bands associated with the presence of CNT at about 1360 cm^−1^ (D band), 1585 cm^−1^ (G band) and 2710 cm^−1^ (2D band) are visible, as it is expected. Apart from them, the bands characteristic for Cu_2_O at about 150 cm^−1^ (T_1u_(TO) mode), 220 cm^−1^ (2E_u_ mode) and 650 cm^−1^ (T_1u_(LO) mode) are found in each of the spectra. This indicates the formation of its crystals within CNT layer during the process of ECD using 1 mM or 100 mM CuSO_4_ solutions. Based on the intensities of the bands observed in the spectra obtained at different depths, it could be concluded that Cu_2_O do not develop any distinctive clusters within the investigated part of the coatings.

### Examination of the CNT layer microstructure by SEM–EDS

The results of Raman analysis indicated the formation of copper oxides within CNT layers after the process of ECD. Therefore, the examination of their microstructure was also performed. For this, the cross sections of the samples were prepared and analyzed by SEM and EDS.

**Figure 11:**
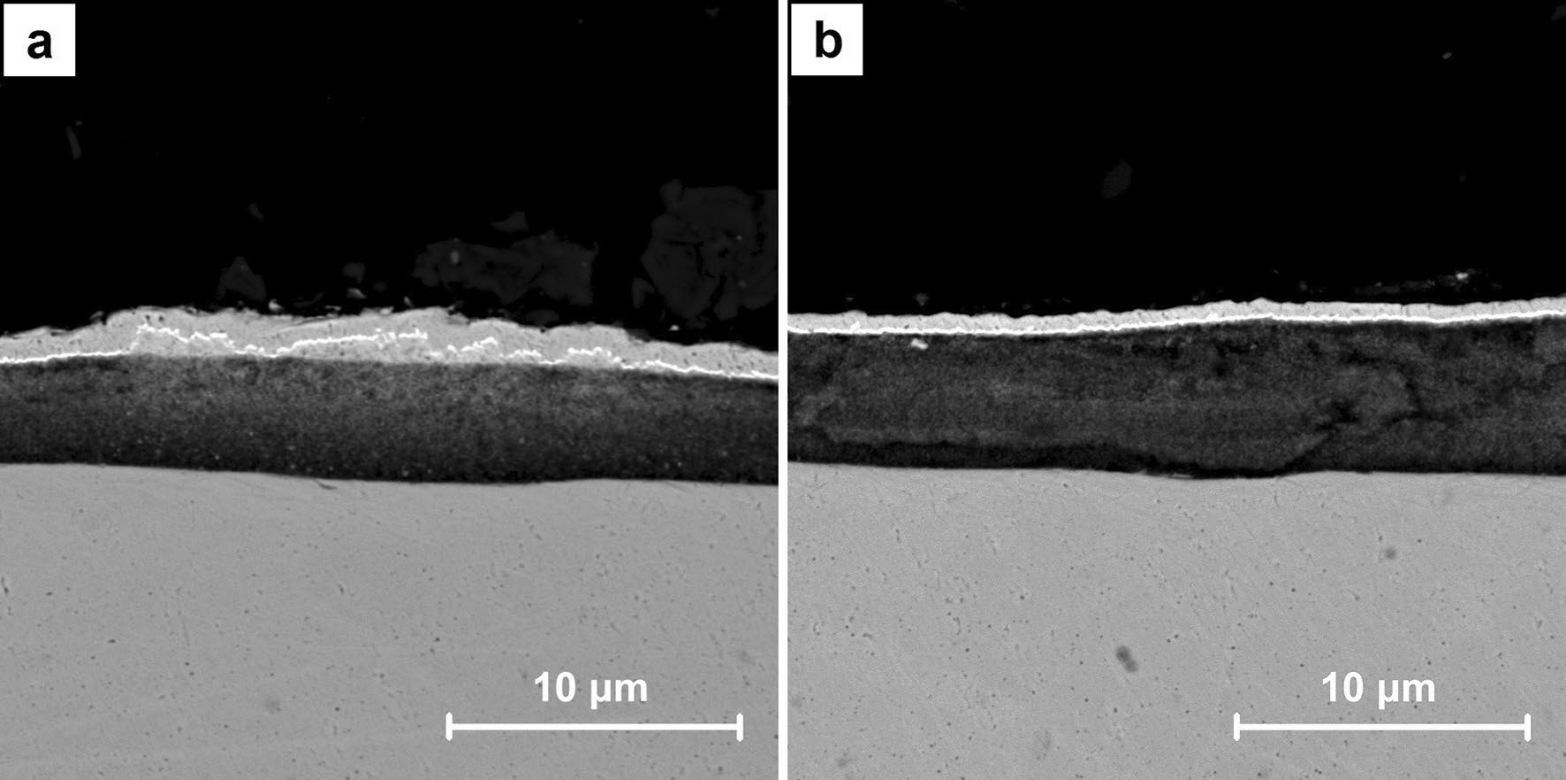
SEM images of the cross sections of A7 sample (ECD deposition time of 600 s, CuSO_4_ concentration of 1 mM) at the magnification of ×10,000.

**Figure 12:**
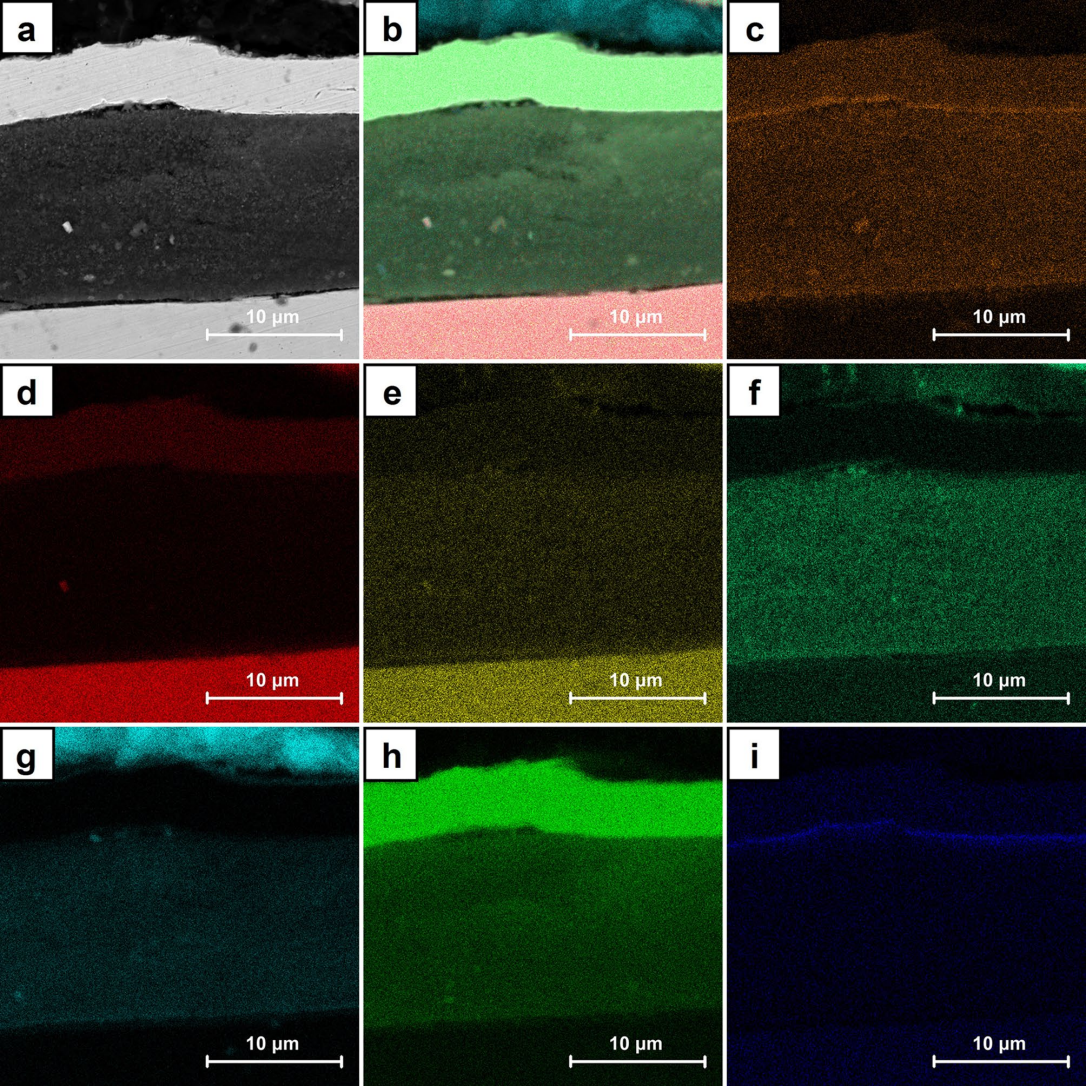
SEM images of A7 sample (ECD deposition time of 600 s, CuSO_4_ concentration of 1 mM): cross section (**a**), cross section with elements distribution (**b**), single element distribution (**c**–**i**) including copper (**c**), iron (**d**), chromium (**e**), oxygen (**f**), carbon (**g**), nickel (**h**) and gold (**i**).

Figure 11 shows the exemplary SEM images for the cross sections of A7 sample. CNT layer with the dendritic Cu crystals on the surface could be identified (Fig. 11a). In its entire volume the nanometric crystals are visible. They are also present in the fragments without Cu deposits on the surface (Fig. 11b). The distributions of the chosen elements obtained by EDS for the cross section of A7 sample are presented in Fig. 12. Chromium and iron are the main components of the stainless steel substrate (Fig. 12d,e) whereas nickel and gold were introduced as the additional layers in preparation for the analysis (Fig. 12h,i). The distribution of copper and oxygen (Fig. 12c,f) within CNT layer confirms the presence of copper oxides and the previously described Raman measurements indicated that this is Cu_2_O. Some small share of the oxygen content could be also associated with the functional groups of CNT introduced during their initial modification.

**Figure 13:**
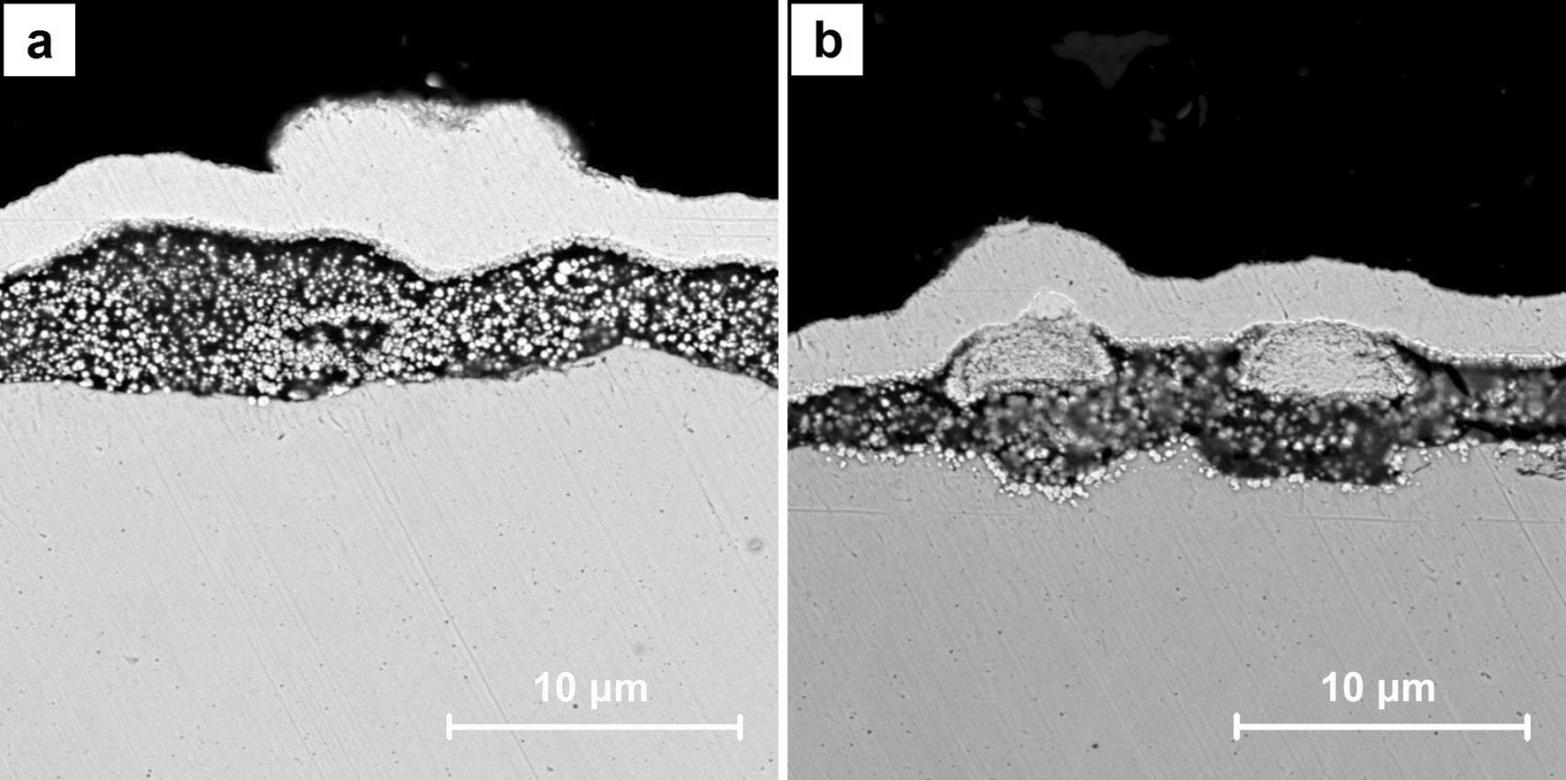
SEM images of the cross sections of B7 sample (ECD deposition time of 600 s, CuSO_4_ concentration of 100 mM) at the magnification of ×10,000.

**Figure 14:**
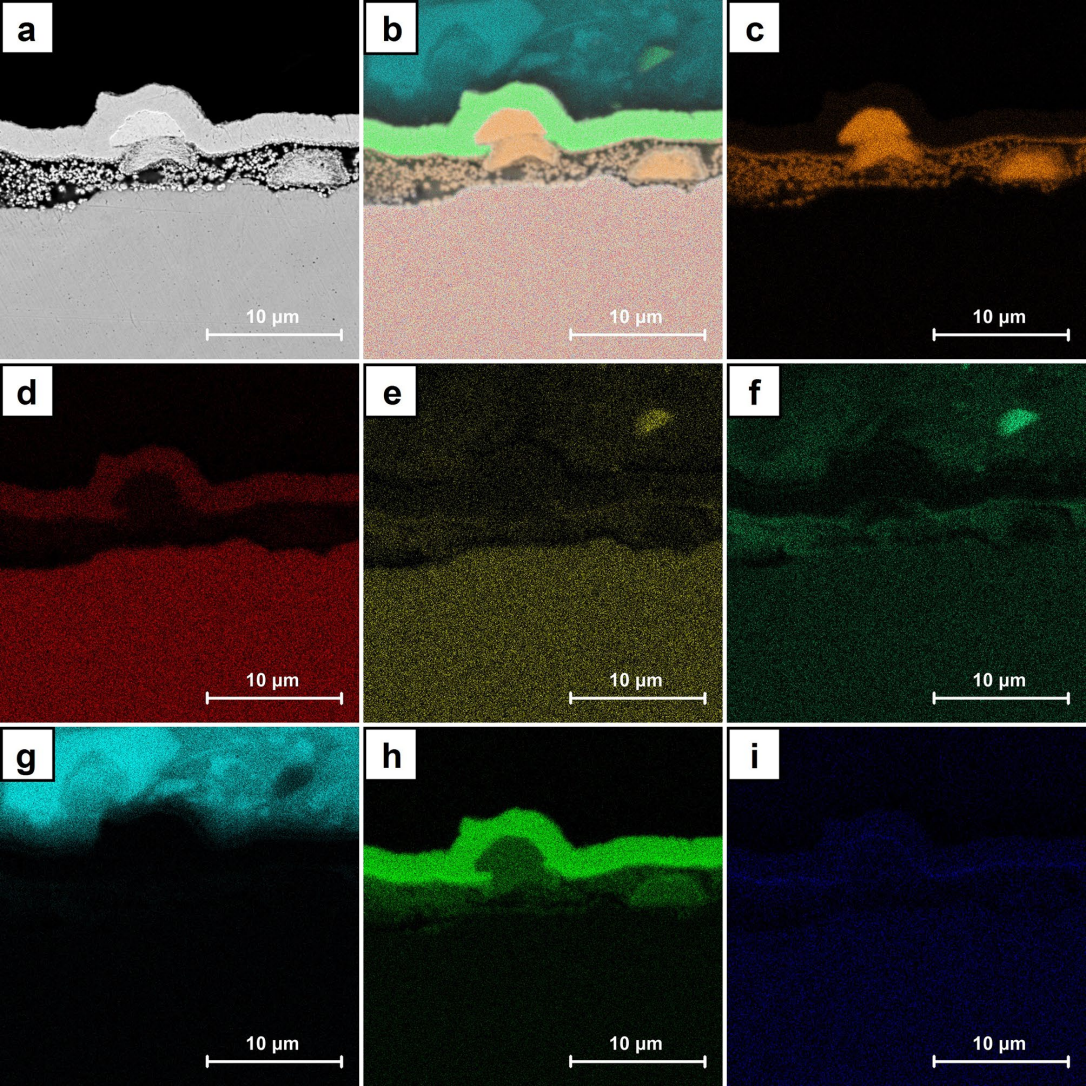
SEM images of B7 sample (ECD deposition time of 600 s, CuSO_4_ concentration of 100 mM): cross section (**a**), cross section with elements distribution (**b**), single element distribution (**c**–**i**) including copper (**c**), iron (**d**), chromium (**e**), oxygen (**f**), carbon (**g**), nickel (**h**) and gold (**i**).

The exemplary SEM images for the cross sections of B7 sample are shown in Fig. 13. In the entire volume of CNT layer, the crystals with dimensions of hundreds of nanometers are observed (Fig. 13a). Therefore, they are significantly bigger than the ones noticed in A7 samples. Some large agglomerates within CNT layer which partially grow to its surface are also identified (Fig. 13b). The others could be formed on the surface or entirely in the volume of CNT layer (Fig. 14a). The distributions of the chosen elements obtained by EDS for the cross section of B7 sample are presented in Fig. 14. The origin of chromium and iron (Fig. 14d,e) as well as nickel and gold (Fig. 14h,i) is the same as for A7 sample. The distribution of copper and oxygen (Fig. 14c,f) within CNT layer also confirms the presence of copper oxides in form of the larger nanometric crystals. Based on the previously described Raman measurements it is known that this is Cu_2_O. Careful analysis of the oxygen distribution also reveals that the large agglomerates are composed only of Cu, but the one on the surface additionally shows the traces of surface oxidation. This result is in accordance with the confocal microscope image (Fig. 7) and the Raman analysis in point B (Fig. 8) which in the similar situation indicates the presence of some amount of Cu_2_O. In the SEM image a local inhomogeneity of CNT layer is observed and explained as related to the crystals growing in its volume. The octahedral Cu_2_O and spherical Cu agglomerates are formed and could grow to the surface. It is known^41,60^ that the thermodynamic deposition potential of metal cations on the same metal substrate is lower in comparison to the one for the substrate composed of the other materials. This is due to the formation of the same continuous crystal lattice by the electrodeposited substance. This effect could be the explanation for the observed Cu agglomerate which grows on the other one partially located within CNT layer (Fig. 14a). When Cu agglomerate grows to the surface it provides a preferred site for the Cu deposition in comparison with pure CNT layer.

### Examination of the CNT layer composition be GIXRD

**Figure 15:**
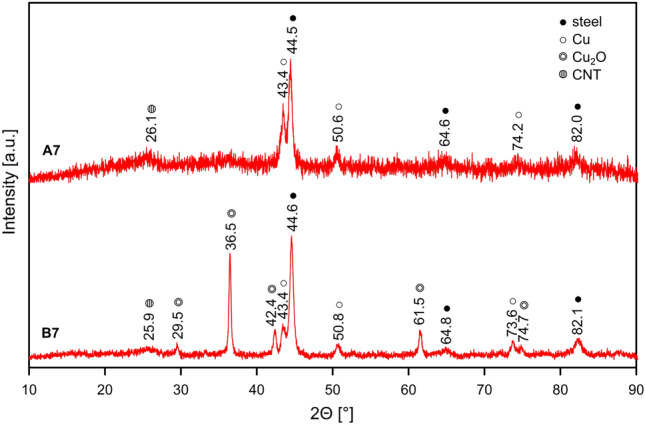
GIXRD patterns for A7 sample (ECD deposition time of 600 s, CuSO_4_ concentration of 1 mM) and B7 sample (ECD deposition time of 600 s, CuSO_4_ concentration of 100 mM).

To support the findings of the SEM–EDS and Raman analysis the GIXRD measurements of the A7 and B7 samples were performed (Fig. 15). This method allows to assess the surface composition of the layers because of the limited penetration depth of X-rays. The diffraction peaks assigned to Cu (COD 4105681) and CNT (COD 1566075) could be identified in both diffractograms, but the ones attributed to Cu_2_O (COD 1000063) may be observed only for B7 sample (Table 3). This is consistent with the previous results and confirms the presence of the octahedral Cu_2_O crystals which grow to the surface of CNT layer during the process of ECD using 100 mM CuSO_4_ solution.

**Table 3:** GIXRD peaks assigned to the components of the coatings in A7 sample (ECD deposition time of 600 s, CuSO_4_ concentration of 1 mM) and B7 sample (ECD deposition time of 600 s, CuSO_4_ concentration of 100 mM).

Cu			Cu_2_O			CNT		
2θ (°)		hkl	2θ (°)		hkl	2θ (°)		hkl
A7	B7		A7	B7		A7	B7	
43.4	43.4	(111)	–	29.5	(110)	26.1	25.9	(002)
50.6	50.8	(200)	–	36.5	(111)	–	–	–
74.2	73.6	(220)	–	42.4	(200)	–	–	–
–	–	–	–	61.5	(220)	–	–	–
–	–	–	–	74.7	(311)	–	–	–

### The mechanism of copper(I) oxide formation within CNT layers

Raman and SEM–EDS analysis revealed the formation of Cu_2_O within CNT layer during the process of ECD. For the samples prepared in the process of ECD using lower concentration of CuSO_4_ solution, i.e. 1 mM (A series), the nanometric crystals were formed whereas using higher concentration of the solution, i.e. 100 mM (B series), the crystals with dimensions of hundreds of nanometers were grown. To explain this effect, we propose the mechanism based on the reaction:

$${{2Cu}^{2+}}_{(aq)}+{2e}^{-}+{H}_{2}O\to {Cu}_{2}{O}_{(s)}+{{2H}^{+}}_{(aq)}$$

It consists of two stages:

$${{Cu}^{2+}}_{(aq)}+{e}^{-}\to {{Cu}^{+}}_{(aq)}$$

$${{2Cu}^{+}}_{(aq)}+{H}_{2}O\to {Cu}_{2}{O}_{(s)}+{{2H}^{+}}_{(aq)}$$

CNT in the layer are functionalized by the initial introduction of the negatively charged functional groups (mainly carboxyl COO^−^ groups). Some of Cu^2+^ cations which diffuse from the bulk solution to their proximity are electrically attracted by them. This bonding enables only the partial reduction of Cu^+^ cations instead of the total reduction to metal Cu. These cations remain bonded to the functionalized CNT. If their local concentration reaches a specific threshold and all the functional groups are occupied, then the crystallization of Cu_2_O occurs. In case of the lower concentration of CuSO_4_ solution, i.e. 1 mM, the diffusion rate significantly decrease. Therefore, the nucleation provides the formation of crystals of smaller size. Contrary, for the higher concentration of the solution, i.e. 100 mM, the increased diffusion rate allows the growth of bigger crystals.

## Conclusions

The high overpotential conditions and the significantly different concentrations of CuSO_4_ solutions applied in the ECD process enabled the formation of various forms of crystals on the surface and within the volume of CNT layer. This indicates that by changing these parameters, the microstructure and chemical composition of the electrodeposited material could be easily controlled. SEM–EDS and Raman spectroscopy, with the addition of GIXRD to confirm the results obtained, were shown as a complete set of methods providing detailed characterization of the samples prepared in this way. Specific attention was given to the formation of crystals within the CNT layer. This required in-depth Raman spectroscopy measurements and SEM–EDS analysis for the cross sections. The study showed that in the high overpotential conditions and using CuSO_4_ solutions with a concentration of 1 mM the formation of dendritic crystals is favored. However, at their concentration of 100 mM, well-defined single crystals are formed. Such differences could be explained by various mechanisms involving the diffusion and reduction during the ECD process. At the lower concentrations of the solutions crystal growth is limited by mass transport, and at the higher concentrations—by the kinetics of the process of cations reduction. A noteworthy phenomenon that was observed for the samples prepared was the formation of Cu_2_O crystals. A mechanism for this process was proposed based on the electrostatic bonding of Cu^2+^ cations by the negatively charged functional groups of CNT, which results only in their partial reduction to Cu^+^ cations. When their local concentration reaches a certain threshold, the crystallization of Cu_2_O occurs. The results obtained may be supportive in the preparation of composite materials based on Cu and CNT with the designed microstructure easily controlled by the ECD process parameters.

## Supplementary Information

Supplementary Information.

## Data Availability

The authors declare that all data supporting the findings of this study are available within the paper and its supplementary information files. Additional data related to this study are available from the corresponding author on reasonable request.
