# Supplementary material for: Characterization of electrochemical deposition of copper and copper(I) oxide on the carbon nanotubes coated stainless steel substrates
Source: Sci Rep. 2023 Apr 26;13:6786. doi: 10.1038/s41598-023-33963-w (PMC10133332; doi:10.1038/s41598-023-33963-w)
Supplement: Supplementary file 1 — Supplementary Information. [file 41598_2023_33963_MOESM1_ESM.docx]

**Supplementary material**


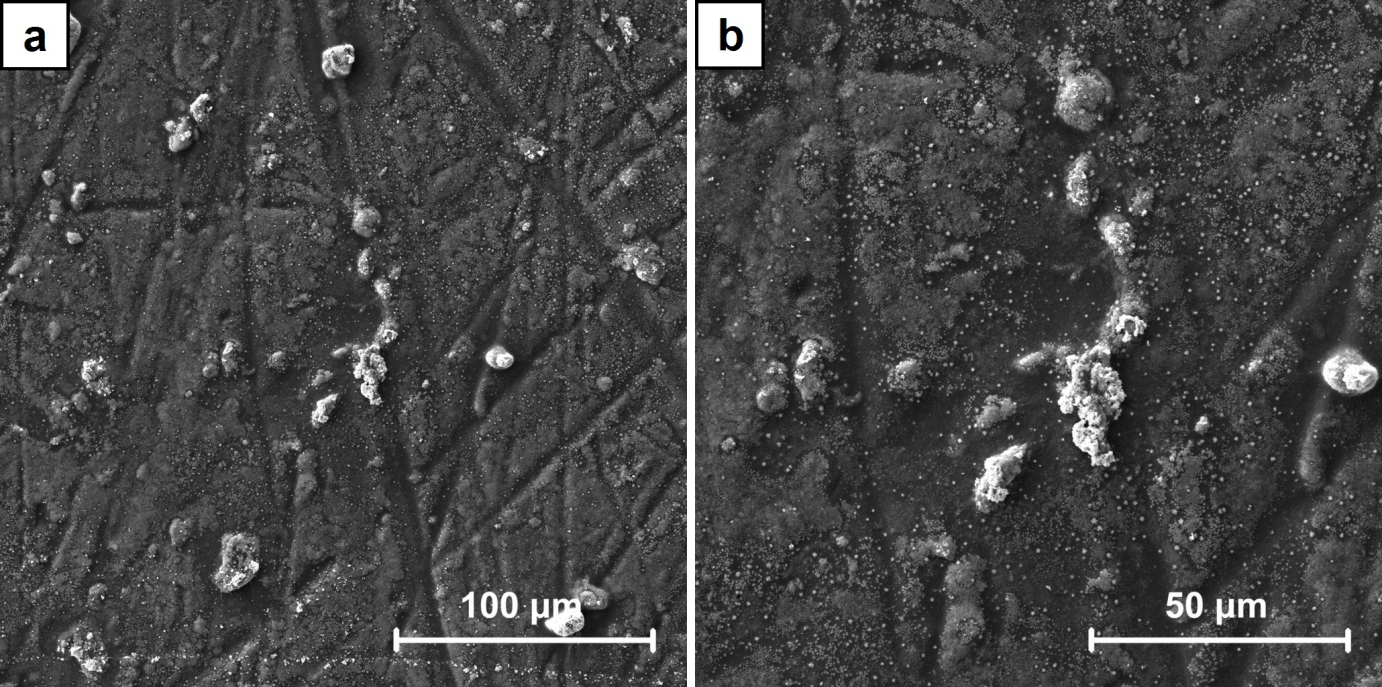


Fig. S1. SEM images of A1 sample at the magnification of 1000x (a) and 2000x (b).


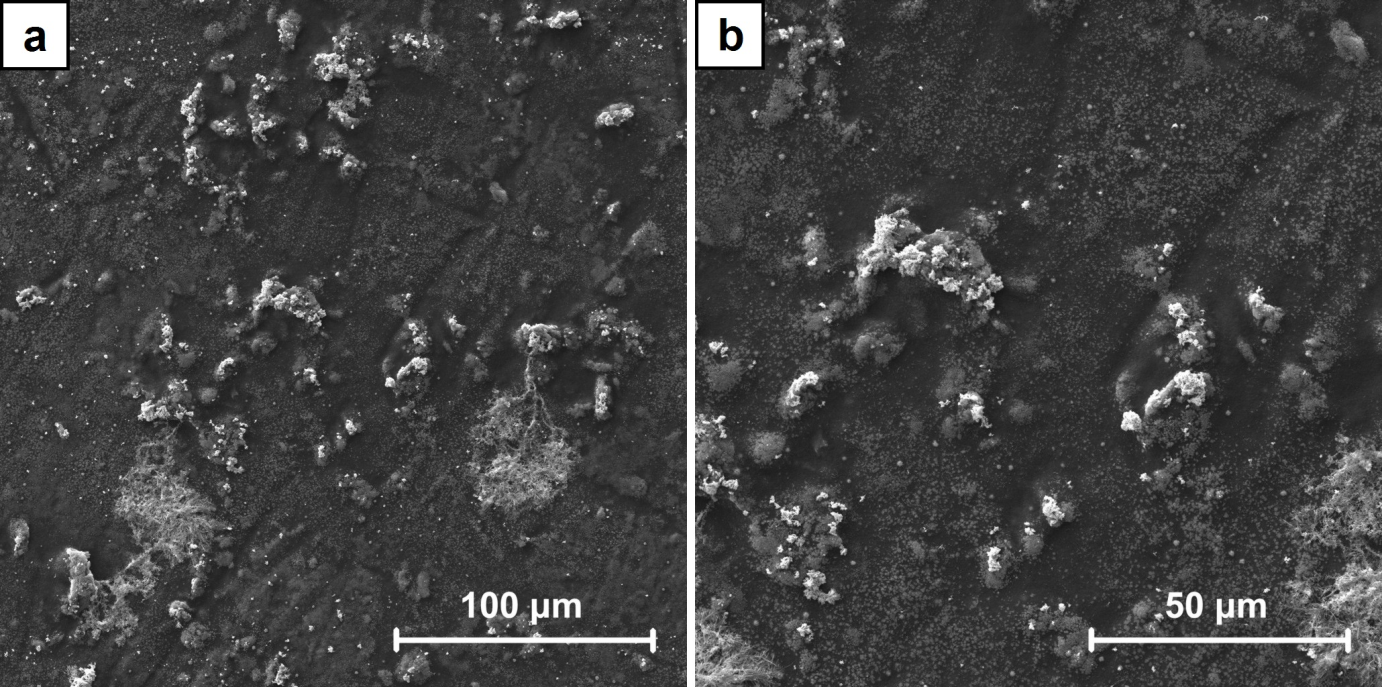


Fig. S2. SEM images of A2 sample at the magnification of 1000x (a) and 2000x (b).


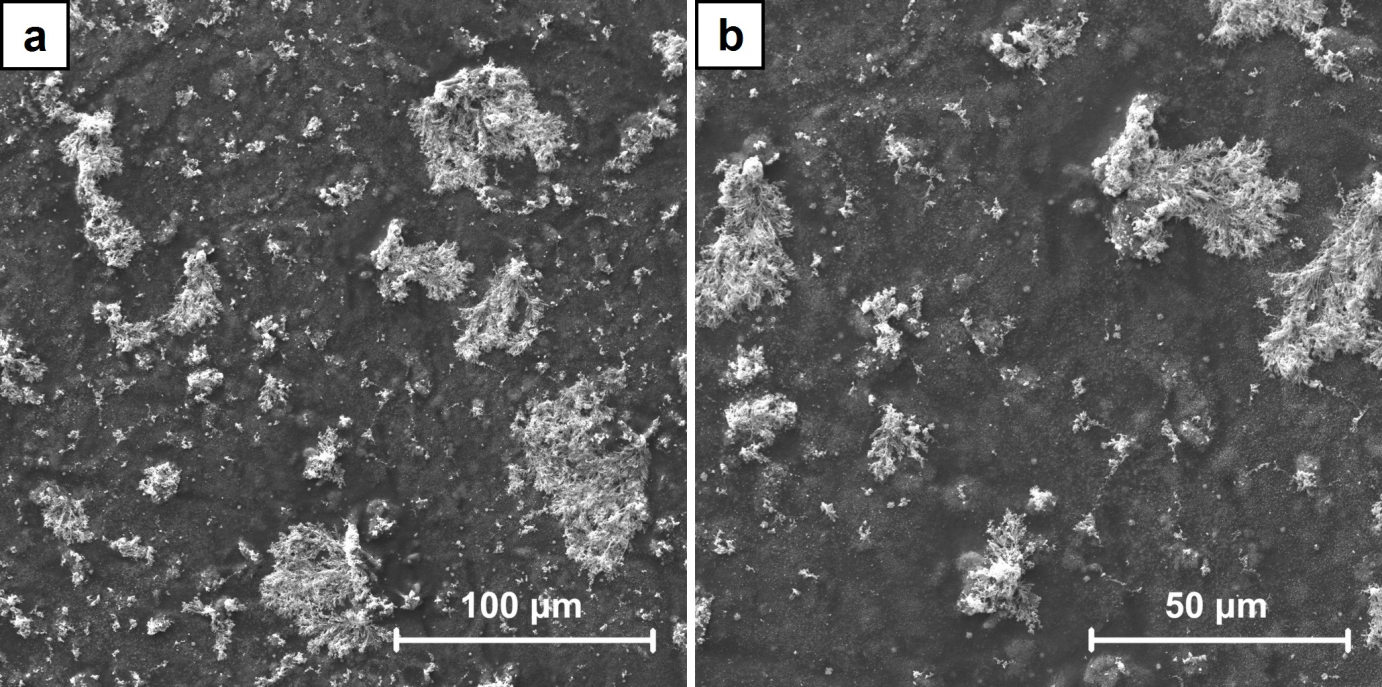


Fig. S3. SEM images of A3 sample at the magnification of 1000x (a) and 2000x (b).


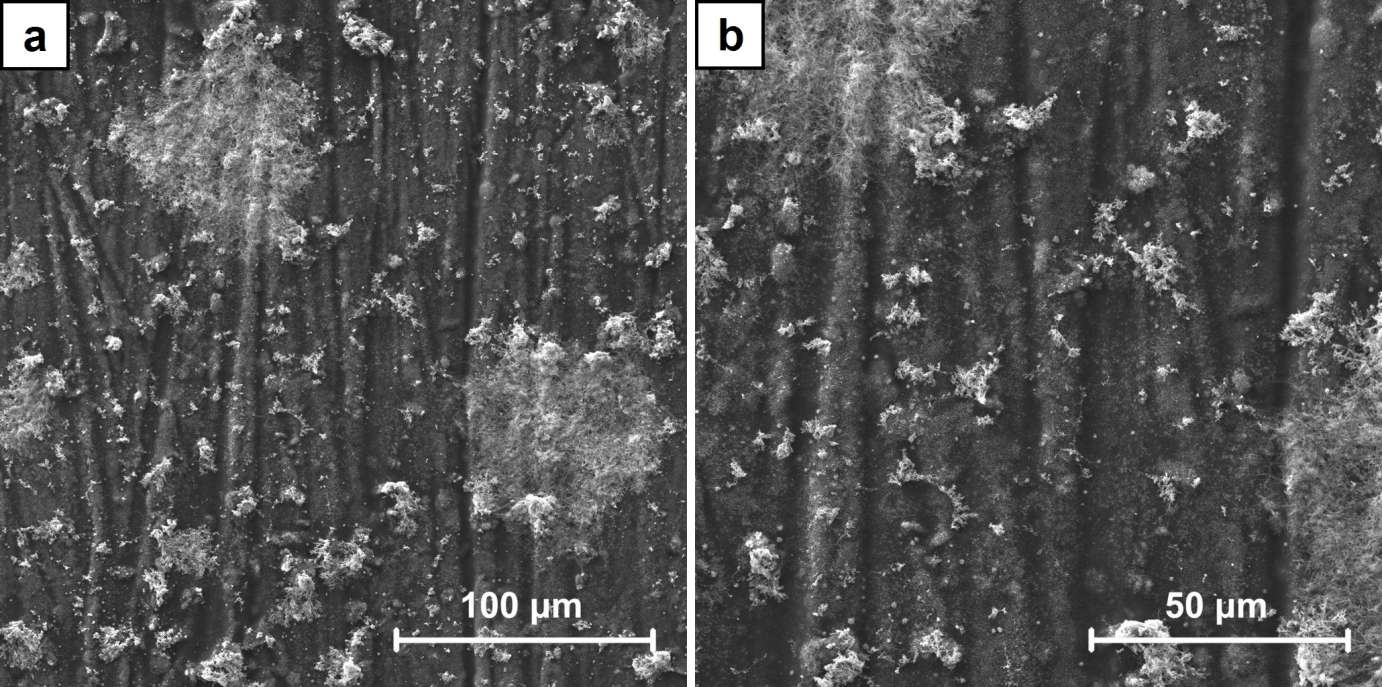


Fig. S4. SEM images of A4 sample at the magnification of 1000x (a) and 2000x (b).


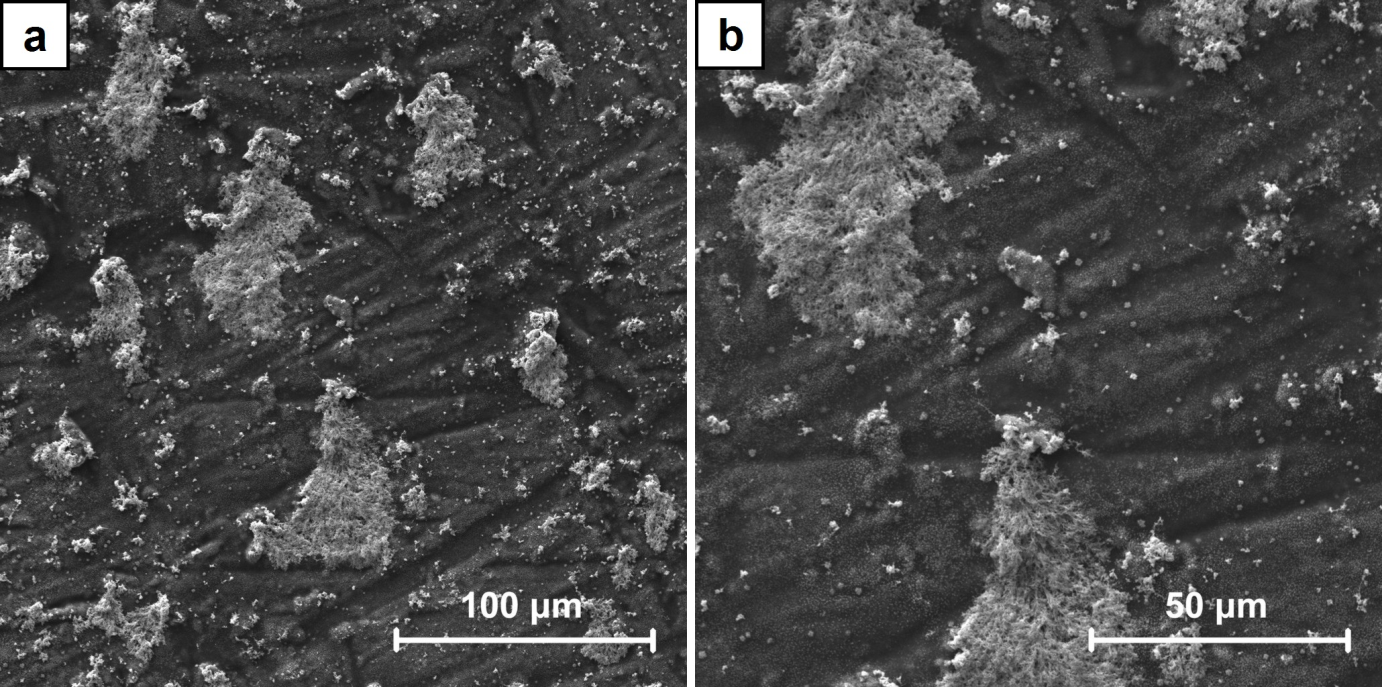


Fig. S5. SEM images of A5 sample at the magnification of 1000x (a) and 2000x (b).


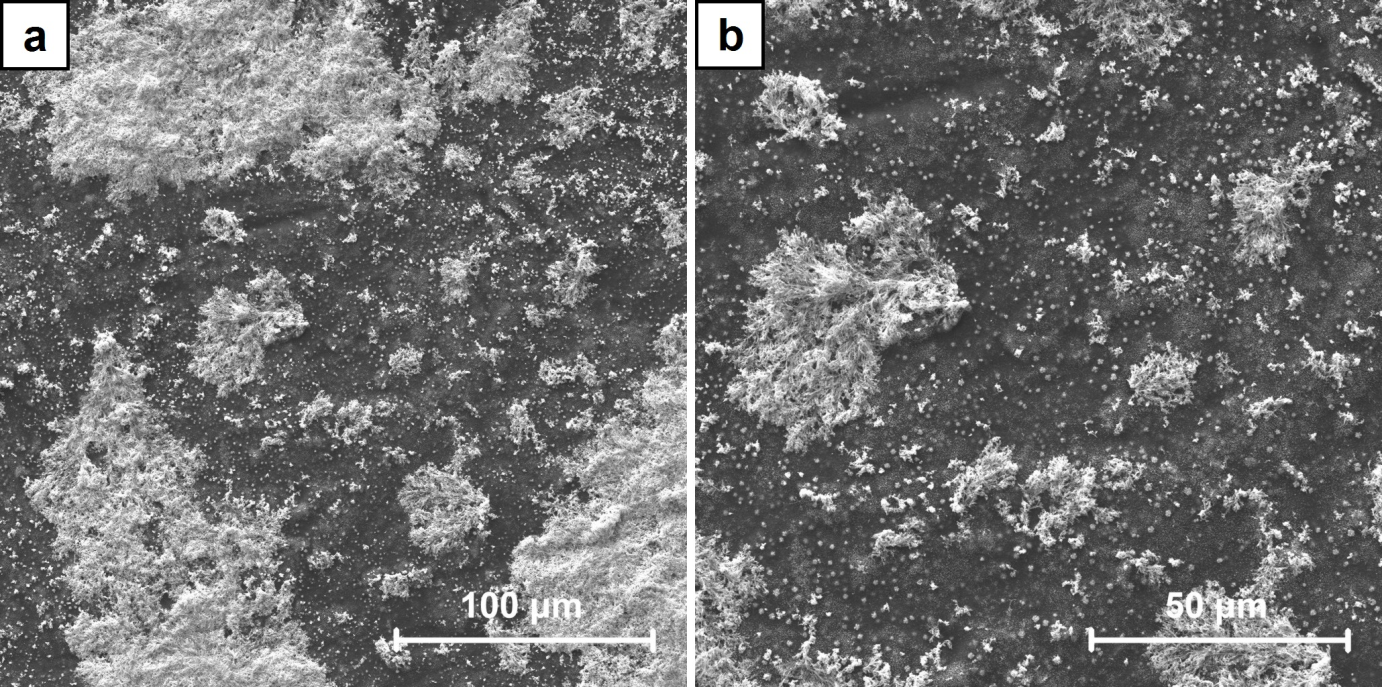


Fig. S6. SEM images of A6 sample at the magnification of 1000x (a) and 2000x (b).


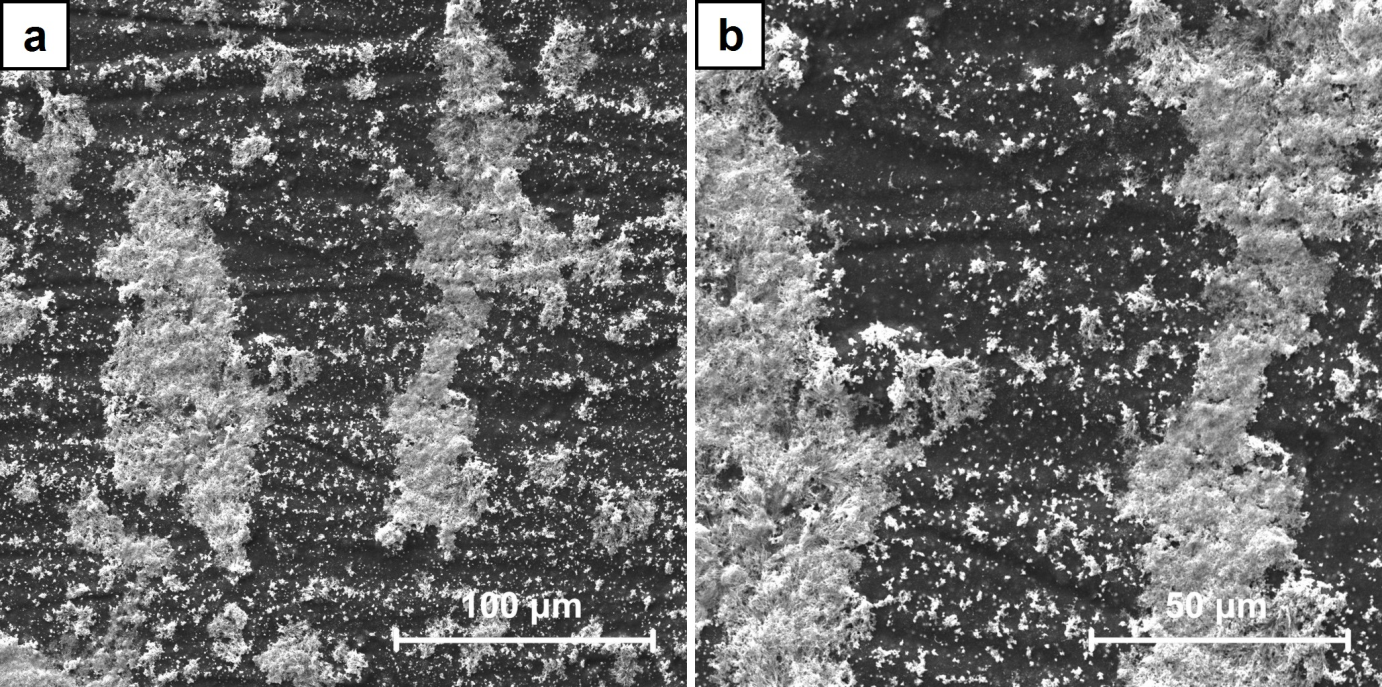


Fig. S7. SEM images of A7 sample at the magnification of 1000x (a) and 2000x (b).


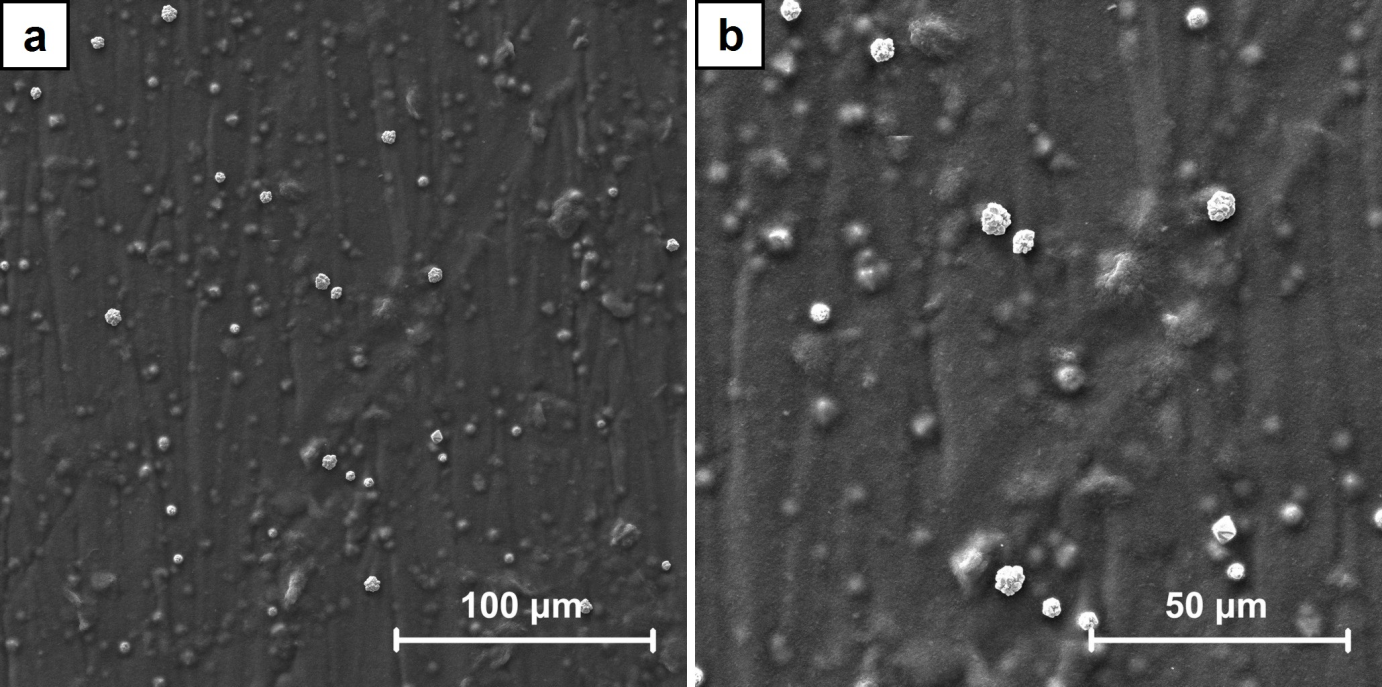


Fig. S8. SEM images of B1 sample at the magnification of 1000x (a) and 2000x (b).


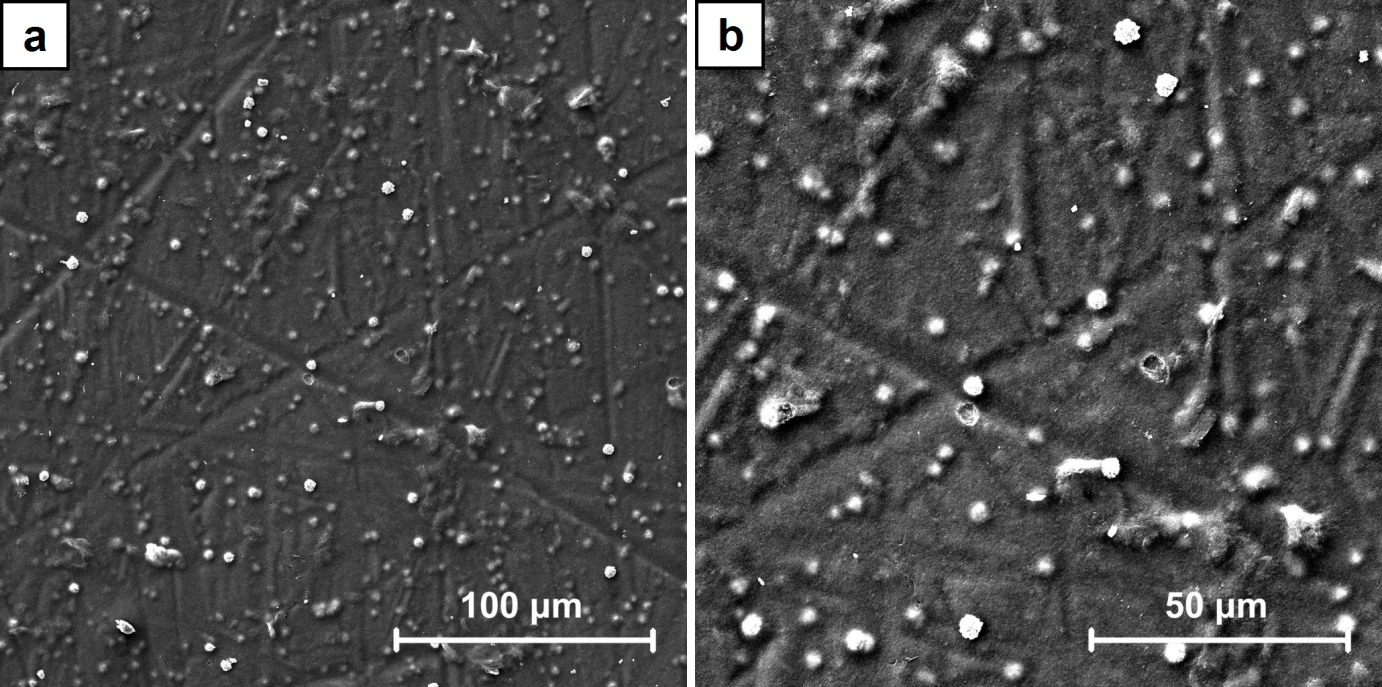


Fig. S9. SEM images of B2 sample at the magnification of 1000x (a) and 2000x (b).


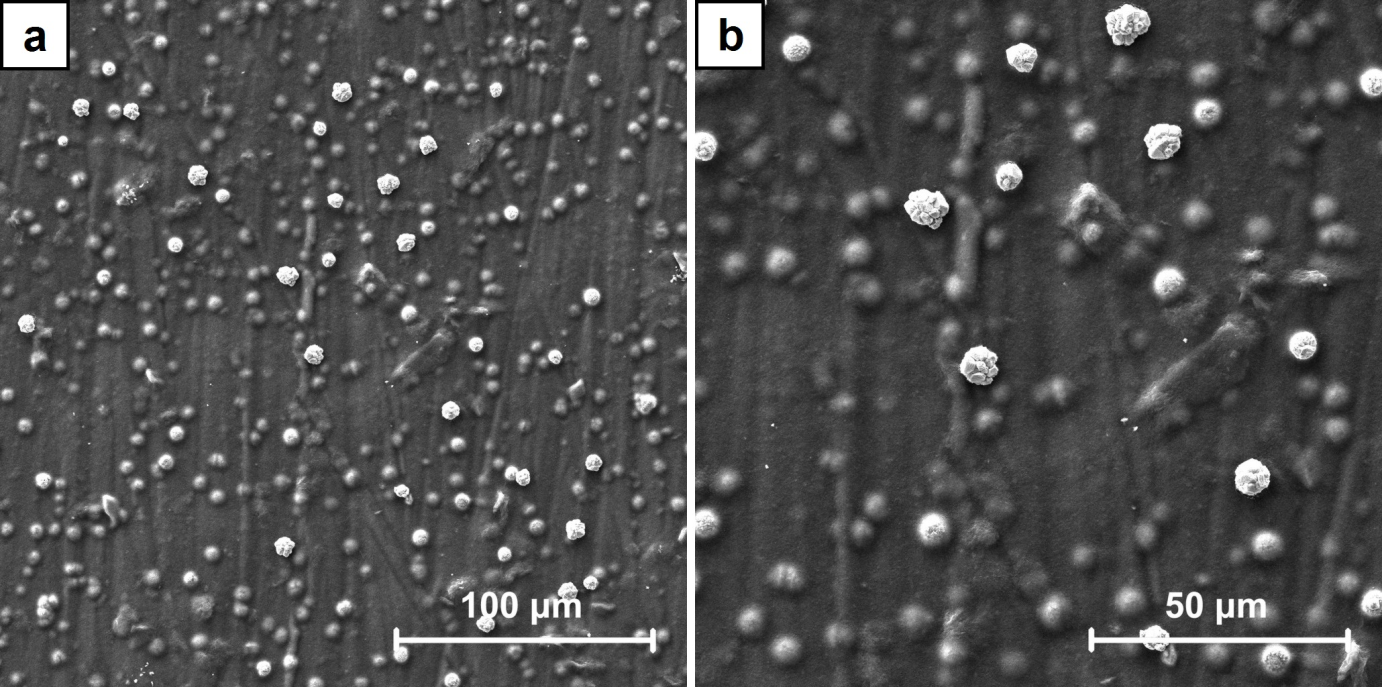


Fig. S10. SEM images of B3 sample at the magnification of 1000x (a) and 2000x (b).


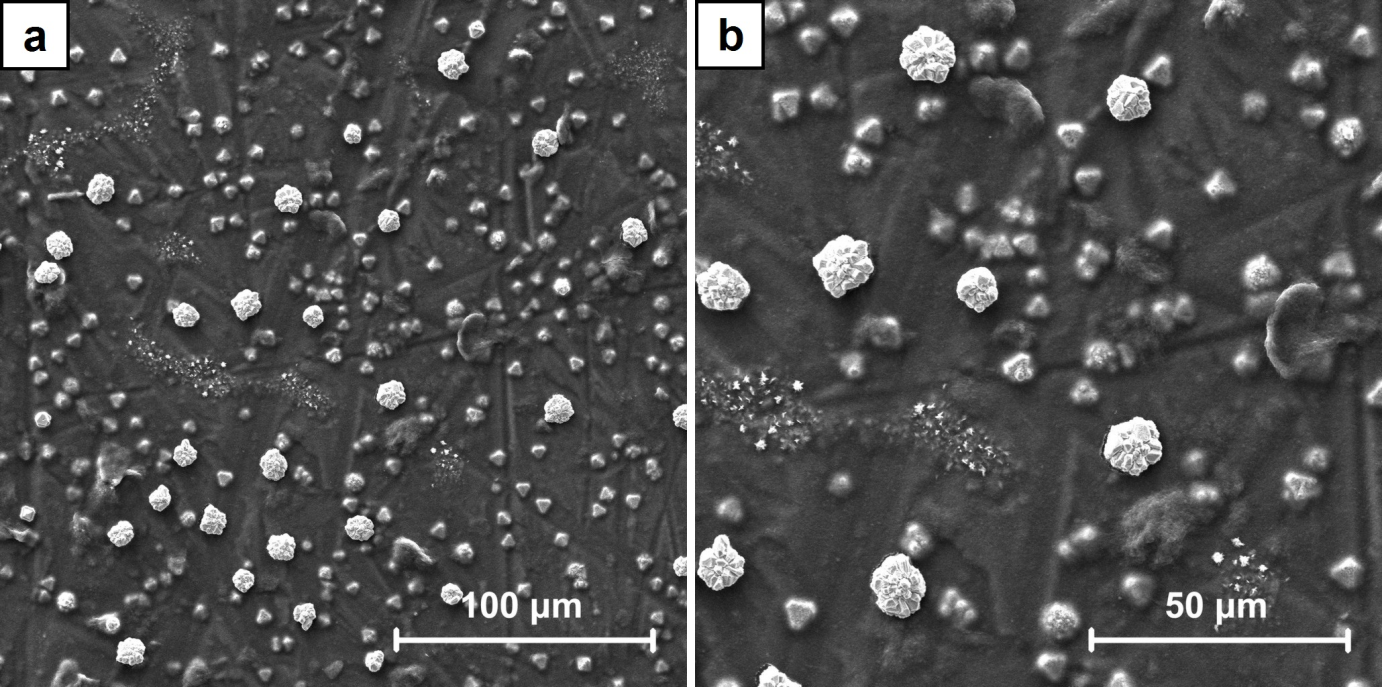


Fig. S11. SEM images of B4 sample at the magnification of 1000x (a) and 2000x (b).


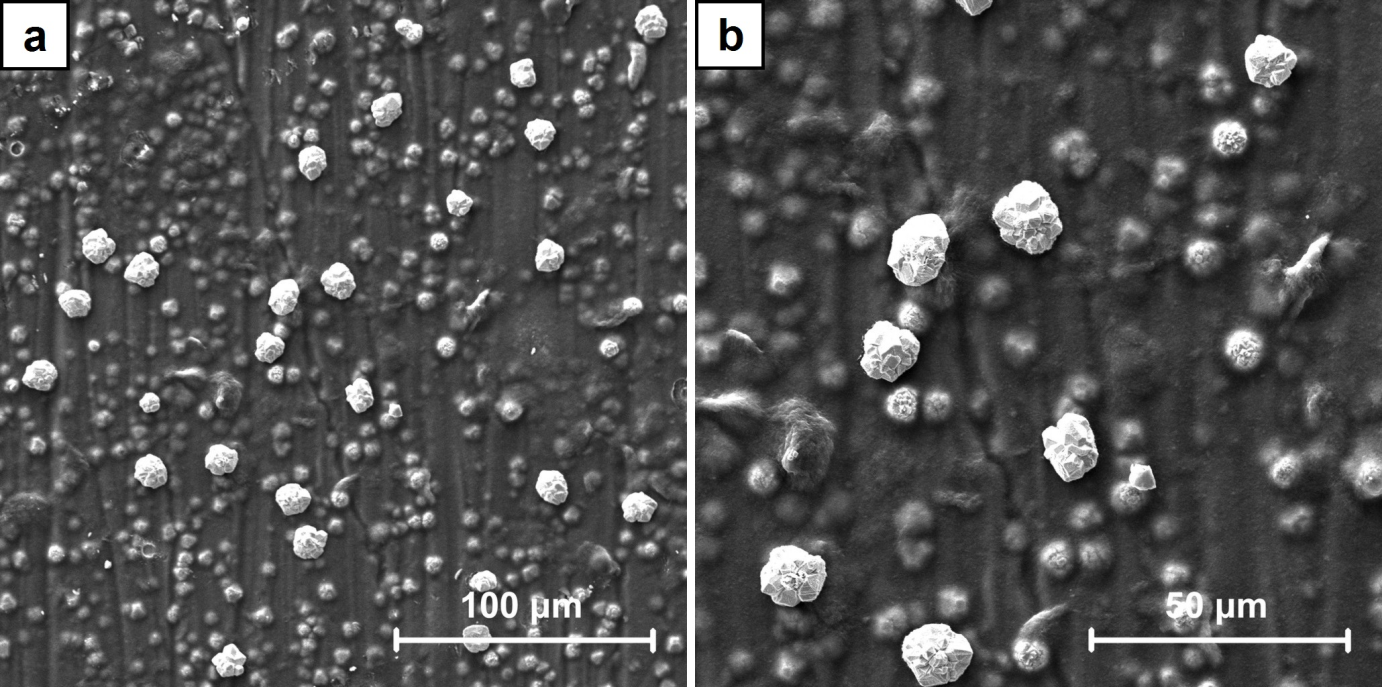


Fig. S12. SEM images of B5 sample at the magnification of 1000x (a) and 2000x (b).


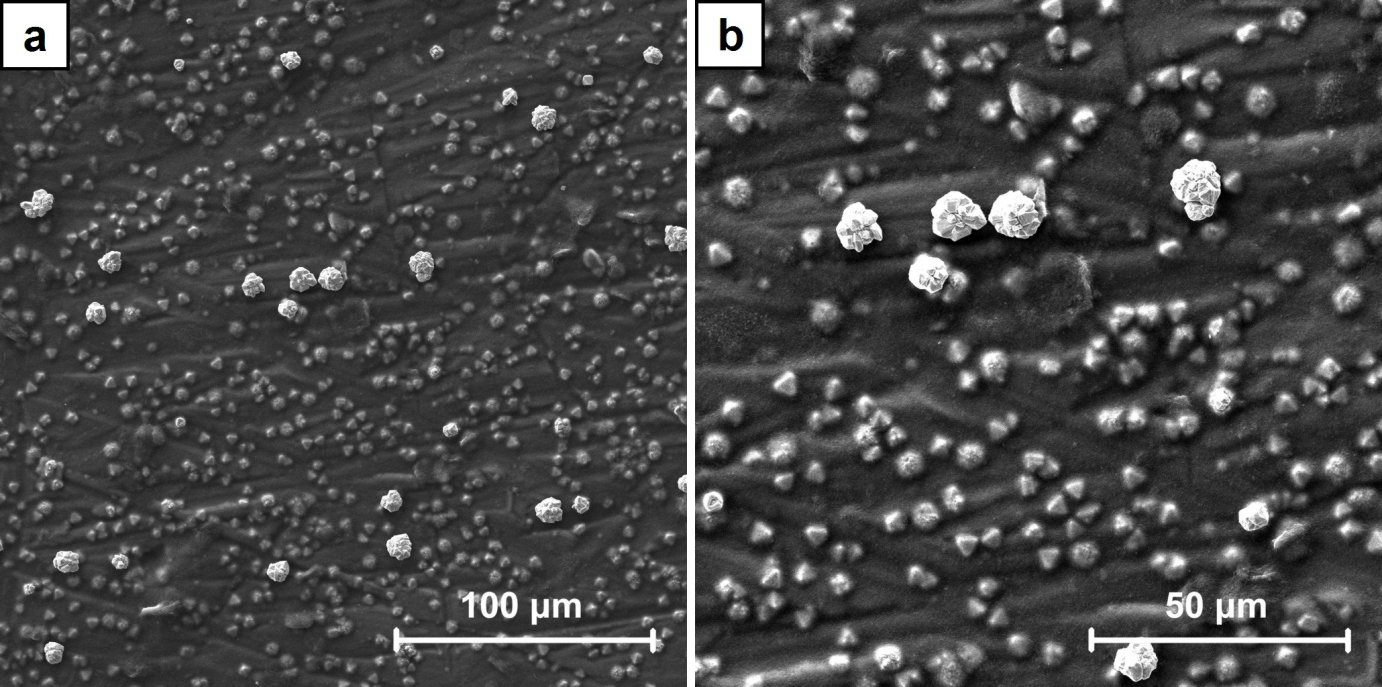


Fig. S13. SEM images of B6 sample at the magnification of 1000x (a) and 2000x (b).


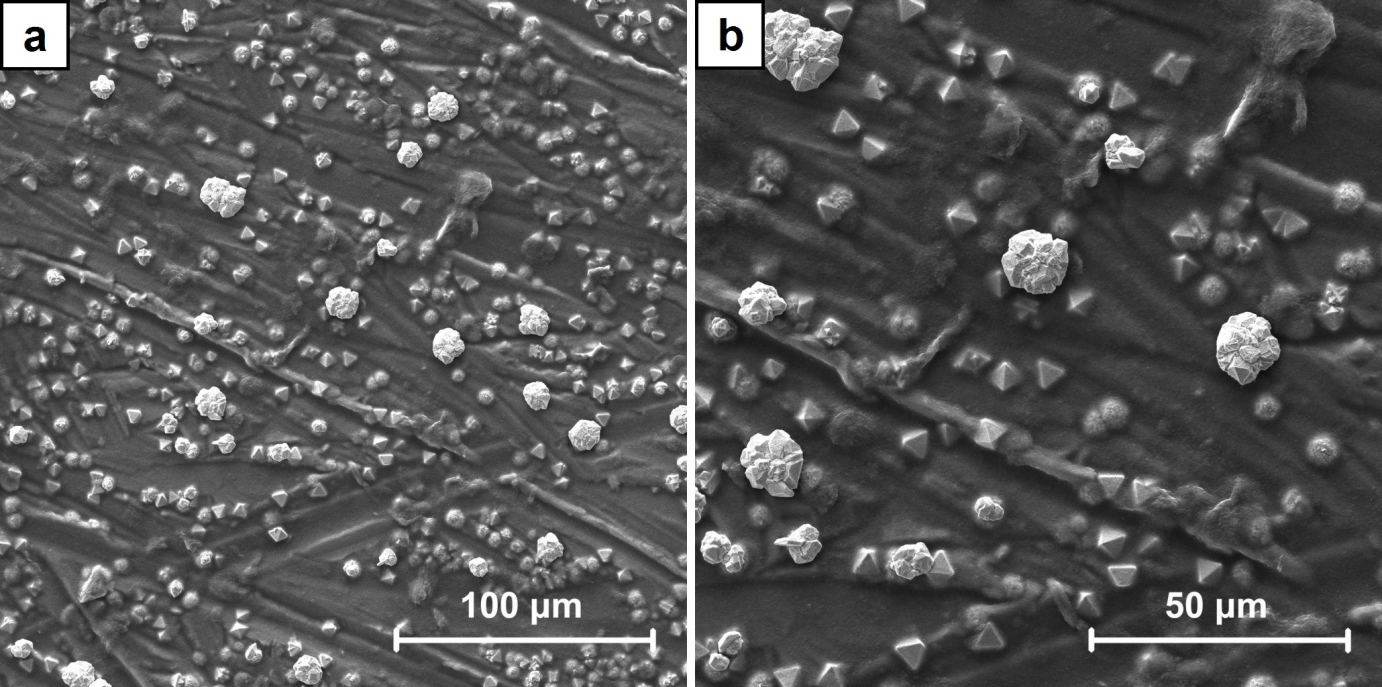


Fig. S14. SEM images of B7 sample at the magnification of 1000x (a) and 2000x (b).


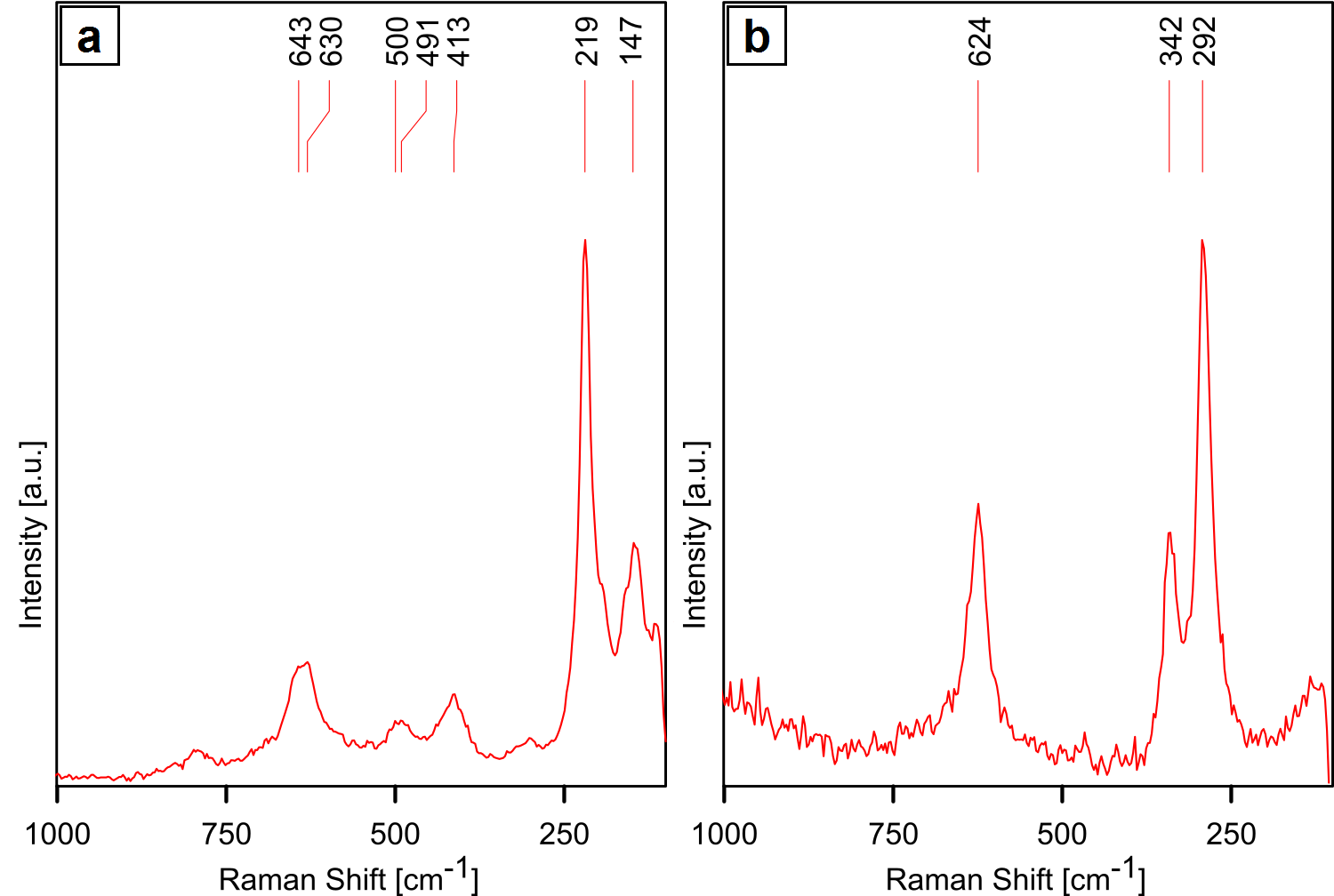


Fig. S15. Raman spectra of the pure Cu_2_O (a) and CuO (b).
